# Phylogenomic analysis of the genus *Leuconostoc*

**DOI:** 10.3389/fmicb.2022.897656

**Published:** 2022-07-25

**Authors:** Stefano Raimondi, Francesco Candeliere, Alberto Amaretti, Stefania Costa, Silvia Vertuani, Gloria Spampinato, Maddalena Rossi

**Affiliations:** ^1^Department of Life Sciences, University of Modena and Reggio Emilia, Modena, Italy; ^2^Biogest Siteia, University of Modena and Reggio Emilia, Reggio Emilia, Italy; ^3^Department of Chemical, Pharmaceutical and Agricultural Sciences—DOCPAS, University of Ferrara, Ferrara, Italy; ^4^Department of Life Sciences and Biotechnology, University of Ferrara, Ferrara, Italy

**Keywords:** *Leuconostoc*, phylogenomics, average nucletide identity (ANI), 16S rRNA gene, cosmeceutics, biopreservatives

## Abstract

*Leuconostoc* is a genus of saccharolytic heterofermentative lactic acid bacteria that inhabit plant-derived matrices and a variety of fermented foods (dairy products, dough, milk, vegetables, and meats), contributing to desired fermentation processes or playing a role in food spoilage. At present, the genus encompasses 17 recognized species. In total, 216 deposited genome sequences of *Leuconostoc* were analyzed, to check the delineation of species and to infer their evolutive genealogy utilizing a minimum evolution tree of Average Nucleotide Identity (ANI) and the core genome alignment. Phylogenomic relationships were compared to those obtained from the analysis of 16S rRNA, *pheS*, and *rpoA* genes. All the phylograms were subjected to split decomposition analysis and their topologies were compared to check the ambiguities in the inferred phylogenesis. The minimum evolution ANI tree exhibited the most similar topology with the core genome tree, while single gene trees were less adherent and provided a weaker phylogenetic signal. In particular, the 16S rRNA gene failed to resolve several bifurcations and *Leuconostoc* species. Based on an ANI threshold of 95%, the organization of the genus *Leuconostoc* could be amended, redefining the boundaries of the species *L. inhae, L. falkenbergense, L. gelidum, L. lactis, L. mesenteroides*, and *L. pseudomesenteroides*. Two strains currently recognized as *L. mesenteroides* were split into a separate lineage representing a putative species (G16), phylogenetically related to both *L. mesenteroides* (G18) and *L. suionicum* (G17). Differences among the four subspecies of *L. mesenteroides* were not pinpointed by ANI or by the conserved genes. The strains of *L. pseudomesenteroides* were ascribed to two putative species, G13 and G14, the former including also all the strains presently belonging to *L. falkenbergense. L. lactis* was split into two phylogenetically related lineages, G9 and G10, putatively corresponding to separate species and both including subgroups that may correspond to subspecies. The species *L. gelidum* and *L. gasicomitatum* were closely related but separated into different species, the latter including also *L. inhae* strains. These results, integrating information of ANI, core genome, and housekeeping genes, complemented the taxonomic delineation with solid information on the phylogenetic lineages evolved within the genus *Leuconostoc*.

## Introduction

The present project aims to investigate the evolutionary relationships within the genus *Leuconostoc* through a phylogenomic approach and to verify the consistency of phylogenetic relationships with the current taxonomy. This genus holds a group of Lactic Acid Bacteria (LAB) belonging, together with the genera *Convivina, Fructobacillus, Oenococcus*, and *Weissella*, to the family of *Leuconostocaceae* (www.bacterio.net; Nieminen et al., 2014; Bello et al., 2022), in its turn included in the order *Lactobacillales*, based on the average amino acid identity of core proteins (cAAI) (Zheng et al., 2020). The genus *Leuconostoc* encompasses 17 species according to the List of Prokaryotic names with Standing in Nomenclature (https://lpsn.dsmz.de/genus/leuconostoc) (Parte et al., 2020). They are saccharolytic bacteria that catabolize carbohydrates to lactic acid through heterolactic fermentation and inhabit a variety of niches where carbohydrate-based substrates are available, such as plants, plant-derived matrices, silage, fermented foods (e.g., dairy products, fermented dough, milk, vegetables, and meats), spoiled foods, and sewage (Dellaglio et al., 1995). In some cases, species are associated with a specific habitat, such as vegetables (Yu et al., 2020), meats (Candeliere et al., 2021), or other foods (Vedamuthu, 1994). The genus is considered safe products and has been accorded the status of “generally recognized as safe” (GRAS) (Ogier et al., 2008). Fermentation of suitable substrates has attracted attention in recent years to produce antimicrobial extracts (Venegas-Ortega et al., 2019; Ahmadi-Ashtiani et al., 2020; Costa et al., 2021). Strains of *Leuconostoc* spp. have found promising industrial applications in the preparation of biopreservative systems by fermentation of different substrates. *Leuconostoc* are used as starters in food and beverage fermentation to improve the nutritional and sensorial properties and to extend the shelf life (Shin and Han, 2015). Moreover, bioactive extracts obtained from vegetables fermented with *Leuconostoc* spp. found application in the formulation of innovative cosmetics (INCI Name: Leuconostoc/Radish Root Ferment Filtrate) (Ahmadi-Ashtiani et al., 2020).

The early taxonomy of LAB was based on phenotypic and morphological features, then chemotaxonomic criteria such as DNA–DNA hybridization and G+C content became the reference for species assignment (Vandamme et al., 1996). With the advent of phylogenetic taxonomy (Woese and Fox, 1977), the sequence of the gene encoding 16S rRNA turned into the gold standard for taxonomic and phylogenetic analysis (Lane et al., 1985; Stackebrandt and Goebel, 1994; Stackebrandt and Ebers, 2006), and allowed the systematic study of evolutionary relationships among prokaryotes. A phylogenetic tree of a conserved gene that is assumed to be vertically inherited is expected to produce the statistical trend or the real genealogy of evolving entities. However, due to the limited size of individual genes, multiple substitutions, parallel, convergent, or reversal events, and horizontal transfer of DNA, the strength of the phylogenetic signal in single molecules is often too low to infer the evolutionary relationships underlying species differentiation. Therefore, multiple phylogenetic markers have been applied to obtain a well-resolved and informative tree, either by the concatenation of genes, aiming to average their phylogenetic information, or by the corroboration of individual phylogenetic signals (Whelan and Morrison, 2017).

With the advent of high-throughput sequencing, an increasing amount of whole bacterial genomes had been accumulated, thus plenty of information became available to improve the resolution of bacterial diversity and the accuracy of phylogenetic reconstruction. In this context, core genome phylogenesis is widely used to infer phyletic lines in the evolutionary history of prokaryotic species (Stott and Bobay, 2020). The average nucleotide identity (ANI) of the genes shared between two genomes was introduced as the gold standard for the delineation of bacterial species (Richter and Rosselló-Móra, 2009; Chun and Rainey, 2014) and has been recently proposed as a tool to determine statistically supported phylogenies (Gosselin et al., 2022).

In the present study, the taxonomy of 221 *Leuconostoc* genome sequences belonging to 17 species has been preliminarily investigated based on ANI to measure genome similarity. ANI, which provides an average measure of similarity across homologous regions shared by a pair of genomes, is a major metric used for this purpose (Palmer et al., 2020), considering the ANI threshold of 95% for species delineation (Richter and Rosselló-Móra, 2009). The subspecies delimitations were also investigated, considering a threshold of 98% (Minias et al., 2020; Pearce et al., 2021), although it is not recognized as a standard in the taxonomy of prokaryotes. For the strains presenting low ANI values, the Average Aminoacidic Identity (AAI) was utilized to confirm the inclusion in the genus *Leuconostoc* and the different putative species, considering the threshold of 55–60% and 85–90%, respectively (Rodriguez-R and Konstantinidis, 2014). The evolutive genealogy of the genomes was reconstructed utilizing a minimum evolution tree of ANI, computed according to Gosselin et al. (2022), and the core genome alignment.

Phylogenetic relationships were also inferred using the housekeeping genes encoding phenylalanyl-tRNA synthase alpha-subunit (*pheS*) and RNA polymerase alpha-subunit (*rpoA*) (Das et al., 2014), in addition to the 16S rRNA gene. *rpoA* was previously exploited for the identification of *L. falkenbergense* strains at the species level (Wu and Gu, 2021a), while *pheS* is one of the targets in the Multilocus Sequence Analysis (MLSA), successfully applied for differentiation of species of the genus *Leuconostoc* (Rahkila et al., 2014). As a whole, multiple phylogenomic approaches have been applied to investigate evolutionary relationships within the genus *Leuconostoc*, following a preliminary reclassification of the strains in putative species according to ANI comparison. Split decomposition analysis has been carried out to check the ambiguities in the inferred phylogenesis, evaluate consistency among the output of the diverse analysis, and determine the approach that provides the strongest phylogenetic signal (Whelan and Morrison, 2017).

## Methods

In total, 221 *Leuconostoc* genome sequences available on 1 October 2021 were retrieved from the NCBI database. The accession numbers are reported in Supplementary Table S1. Only genomes obtained from pure culture sequencing were used, while metagenome-assembled genomes were discarded. The genomes were inspected for completeness and contamination with CheckM (Parks et al., 2015). Genomes were also checked for the presence of the molecular signatures characterizing the genus *Leuconostoc* (Bello et al., 2022). The genomes were annotated with Prokka (Seemann, 2014) to calculate the pangenome using Roary (Page et al., 2015), with the minimum percentage identity parameter set at 80%.

The genes encoding *rpoA, pheS*, and 16S rRNA were extracted from each genome and aligned with Clustal Omega on EMBL-EBI website (Sievers et al., 2011; Madeira et al., 2019). The Total Average Nucleotide Identity (ANI) was calculated following the method described by Gosselin et al. (2022). This method allowed us to calculate the bootstrap values from 100 ANI distance matrices replicates, thanks to a process of genome segmentation and random segment selection utilized to calculate the desired number of replicates. The AAI between all the genomes was calculated with AAI Calculator (http://enve-omics.ce.gatech.edu/aai/) by all-against-all approach (Rodriguez-R and Konstantinidis, 2016).

The alignments of 16S rRNA, *rpoA*, and *pheS* genes and the 247 core genes identified by Roary out of a total of 25,377 genes (Supplementary Spreadsheet 1) were utilized to generate phylogenetic trees constructed with the maximum likelihood method using the RAxML tool with 100 bootstrap replicates (Stamatakis, 2014). The ANI distance matrix was used to build a tree using the script provided by Gosselin et al. (2022). In this script, the balanced minimum evolution algorithm implemented in the FastME function of the R package APE (Paradis et al., 2004) was applied to generate phylogenies for each distance matrix (Desper and Gascuel, 2002), whereas the function *plotBS* of the R package Phangorn (Schliep, 2011) was exploited to map support values onto the tree. The AAI distance matrix was used to compute an Unweighted Pair Group Method with the Arithmetic mean (UPGMA) unrooted phylogenetic tree with the DendroUPGMA tool (Garcia-Vallvé et al., 1999). The phylogeny was further inferred with SplitsTree v. 4.18.2 (Huson and Bryant, 2006) with a neighbor net drawing and Jukes–Cantor correction for alignment-derived trees (Bandelt and Dress, 1992; Huson and Bryant, 2006).

The tree of ANI was compared with those constructed from the alignment of 16S rRNA, *pheS, rpoA* genes, or of the core genome, utilizing the following “generalized” Robinson–Foulds metrics: the Jaccard–Robinson–Foulds (JRF), computed with *k* = 1 (Nye et al., 2006), and the information-based measures of Mutual Clustering Information (MCI) and Shared Phylogenetic Information (SPI) (Smith, 2020). JRF, MCI, and SPI were computed with the R package “TreeDist,” archived at https://dx.doi.org/10.5281/zenodo.3528123. Comparison relied on matching each informative split within a tree (i.e., an internal branch of the tree, with at least two leaves at each extremity) with an informative split within the other tree. For each metric, the comparison yielded a normalized similarity score in the range 0–1 between two trees and a score for each match of paired tree splits.

## Results

### ANI phylogenomic analysis

All the genome sequences available for species of *Leuconostoc* on the date 1 October 2021, containing all the type strains, were included in the analysis. The majority of the sequences presented low or no contamination (≤ 5%) after CheckM analysis, except for six genomes. Five of them were discarded, while the sequence of *L. inhae* KCTC 3774 (contamination = 6.1%) was retained, being a type strain. As a whole, 216 genomes were analyzed. They were ascribed to 17 correctly named species (17 *L. carnosum*, 29 *L. citreum*, three *L. falkenbergense*, two *L. fallax*, three *L. gelidum*, seven *L. gasicomitatum*, one *L. holzapfelii*, five *L. inhae*, two *L. kimchii*, 19 *L. lactis*, one *L. litchii*, one *L. miyukkimchii*, 83 *L. mesenteroides*, one *L. palmae*, 34 *L. pseudomesenteroides*, one *L. rapi*, and six *L. suionicum*), to the not validly published species “*L. garlicum”* (one strain), and broadly assigned to the genus *Leuconostoc* (five strains) (Supplementary Table S1). All the strains, including those without an assigned species, were coherent with the indel signatures of *Leuconostoc* genus.

**Figure 1:**
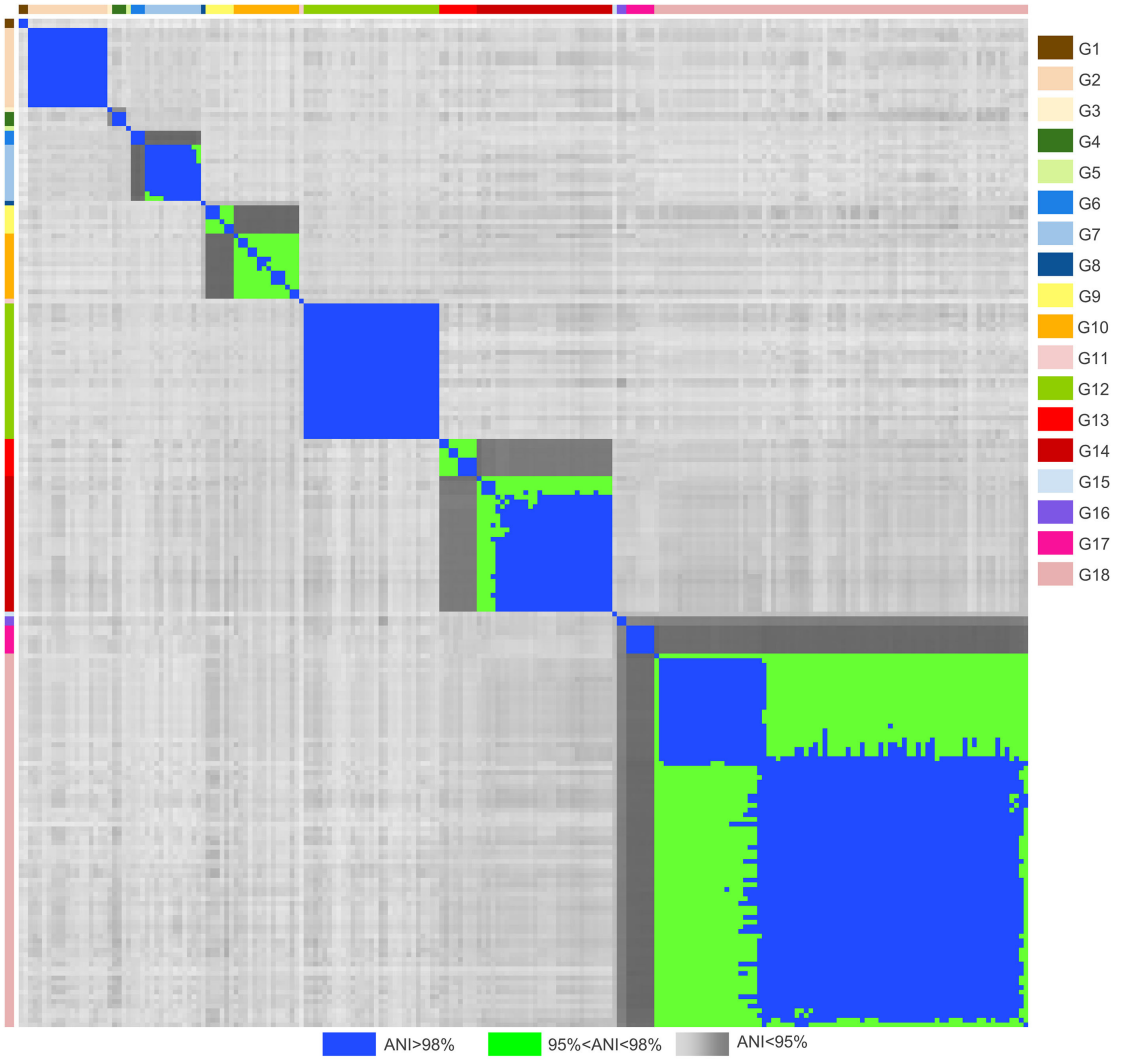
Heatmap of the ANI similarities matrix reporting pairwise ANI values of 216 *Leuconostoc* genomes. Blue, ANI > 98%; green, 95% < ANI <98%; gray shades, ANI <95% (77–94.9%). Strain labels are colored according to the groups.

**Figure 2:**
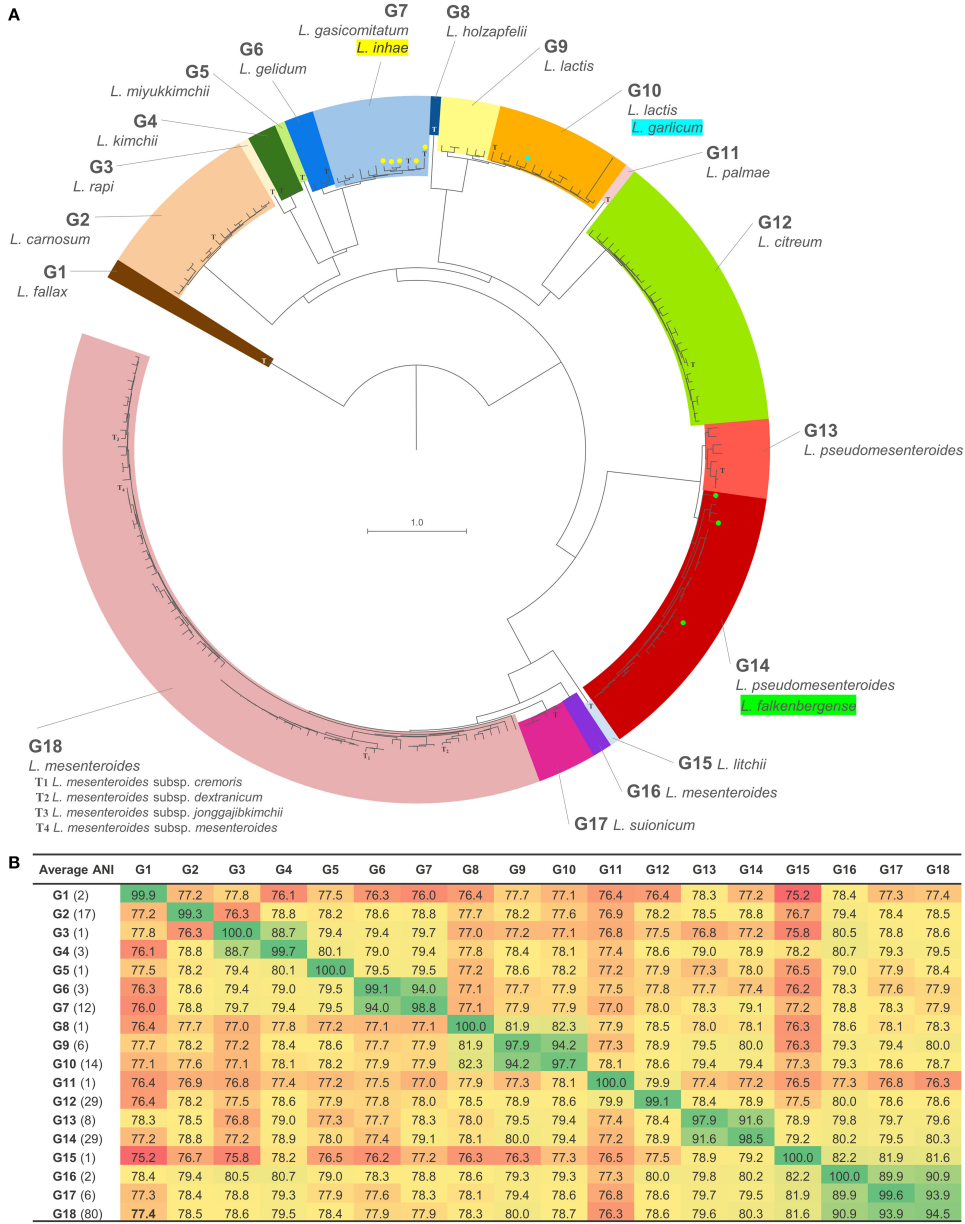
Phylogenomic analysis of *Leuconostoc* species: **(A)** Minimum evolution tree of ANI, reporting the 18 proposed clades with values >95%; T, type strain; strains currently ascribed to the species of *L. falkenbergense, L. garlicum*, and *L. inhae* are indicated in green, cyan, and yellow, respectively. **(B)** Heatmap of the mean ANI values between the strains of the groups.

Pairwise ANI values were calculated for the data set (Figure 1, Supplementary Spreadsheet S2). Considering the species threshold of 95%, the 216 strains nominally ascribed to 17 species were redistributed in 18 groups (referred to as G1–G18), which were consistent with the taxonomy of the Genome Taxonomy Database web server (GTDB, https://gtdb.ecogenomic.org; Supplementary Table S1). Pairwise ANI between strains of different groups was always <95%, while it was always >6% between strains belonging to the same group, thus ascribable to the same putative species (Figures 1, 2A). The mean pairwise ANI similarities between the strains of each group are presented in Figure 2B.

The species *L. fallax, L. carnosum, L. rapi, L. kimchii, L. miyukkimchii, L. holzapfelii, L. citreum, L. palmae, L. litchii*, and *L. suionicum* were consistent with the current taxonomy and coincided with groups G1, G2, G3, G4, G5, G8, G11, G12, G15, and G17, respectively. Except for a sole strain of *L. citreum*, none of the strains attributed to these species was included in a different group. On the other hand, a bijective relationship between nominal species and ANI groups (i.e., one species–one group, and *vice versa*) was not observed for the strains currently belonging to *L. mesenteroides, L. pseudomesenteroides, L. falkenbergense, L. lactis, L. inhae*, and *L. gelidum*. The five strains lacking a nominal species designation were placed in groups G4, G12, G13, G14, and G18 (Supplementary Table S1).

Most of the strains assigned to the species *L. mesenteroides* (80) clustered in G18. This group encompassed all the strains belonging to the four validly published *L. mesenteroides* subspecies (*L. mesenteroides* subsp. *cremoris, L. mesenteroides* subsp. *dextranicum, L. mesenteroides* subsp. *mesenteroides*, and *L. mesenteroides* subsp. *jonggajibkimchii*), including the corresponding type strains (ATCC 19254^T^, DSM 20484^T^, ATCC 8293^T^, and DRC1506^T^, respectively), and to the not valid subspecies *L. mesenteroides* subsp. *sake*. Within G18, the strains were very similar and, despite the existence of different nominal subspecies, they presented on average ANI values of 99.1%. Pairwise ANI values between members of the different subspecies of *L. mesenteroides* were always >98.6%. Likely evolving as a separate lineage, only *L. mesenteroides* subsp. *cremoris* seemed fully resolved at the subspecies level (except for *L. mesenteroides* subsp. *cremoris* LbT16, likely misclassified). Another strain of *L. mesenteroides* and *L. mesenteroides* subsp. *mesenteroides* clustered in a diverse clade, G16, which are closely related to both G17 and G18, the latter grouping the *L. suionicum* strains.

The strains attributed to the species *L. pseudomesenteroides* were distributed in the closely related groups G13 and G14. The mean value of pairwise ANI between strains from the two distinct groups was 91.8%. The type strain *L. pseudomesenteroides* NCDO 768^T^, seven other *L. pseudomesenteroides*, and a *Leuconostoc* sp. were comprehended in G13, with a mean ANI value of 98.4%. G14 held 26 *L. pseudomesenteroides*, three strains currently ascribed to *L. falkenbergense*, including the type strain *L. falkenbergense* LMG 10779^T^, and an additional *Leuconostoc* sp. strain, with a mean ANI value of 99.4%.

The strains belonging to the species *L. lactis* were split into two phylogenetically related groups, G9 and G10. G9 included six *L. lactis* strains, among which *L. lactis* KCTC 3773, which used to be the type strain of the species *L. argentinum* until it was merged with *L. lactis*. G10 encompassed the type strain of *L. lactis* (strain JCM 6123^T^), the putative “L. garlicum” KFRI01 (species currently not validly published), *L. citreum* 1300_LCI^T^, and other 12 *L. lactis*. Pairwise ANI between strains of G9 and G10 was always <94.5%, while it was always >96.9% within the two groups. Within G9, two subgroups of strains with ANI values >98% within them and in the range of 95–98% between the subgroups could be delineated. Likewise, pairs of strains presenting ANI values >98% were identified within G10, even though a clear delineation of subgroups could not be accomplished.

The strains of *L. gelidum* and *L. gasicomitatum* clustered in two separated but closely related clades, corresponding to G6 and G7, the latter also including *L. inhae* (Figure 1). G6 comprised three *L. gelidum* strains, among which the type strain *L. gelidum* KCTC_3527^T^. G7 comprised all the *L. gasicomitatum* (7) and all the *L. inhae* (5), including the corresponding type strain. Within this group, the pairwise ANI values between strains of *L. gasicomitatum* and *L. inhae* were always > 98%.

Phylogenomic relationships highlighted by ANI joined G17 and G18, which were more distantly related to G16 and G15 (Figure 2). Another strict relationship is associated with G13 and G14, which were more remotely linked to the clade harboring G15, G16, G17, and G18. A close relationship connected G9 and G10, which were more distantly related to G8, with these three groups descending from a common branch that evolved independently also toward G11 and G12. Strict relationships were found between G3 and G4 and between G6 and G7, which lay in a clade harboring also G5, and with a more ancient bifurcation, G2. On the other side, group G1 resulted phylogenetically distant from all the other putative species, albeit belonging to the genus *Leuconostoc* according to ANI thresholds.

### Comparison between ANI and phylogenetic genomic markers

**Figure 3:**
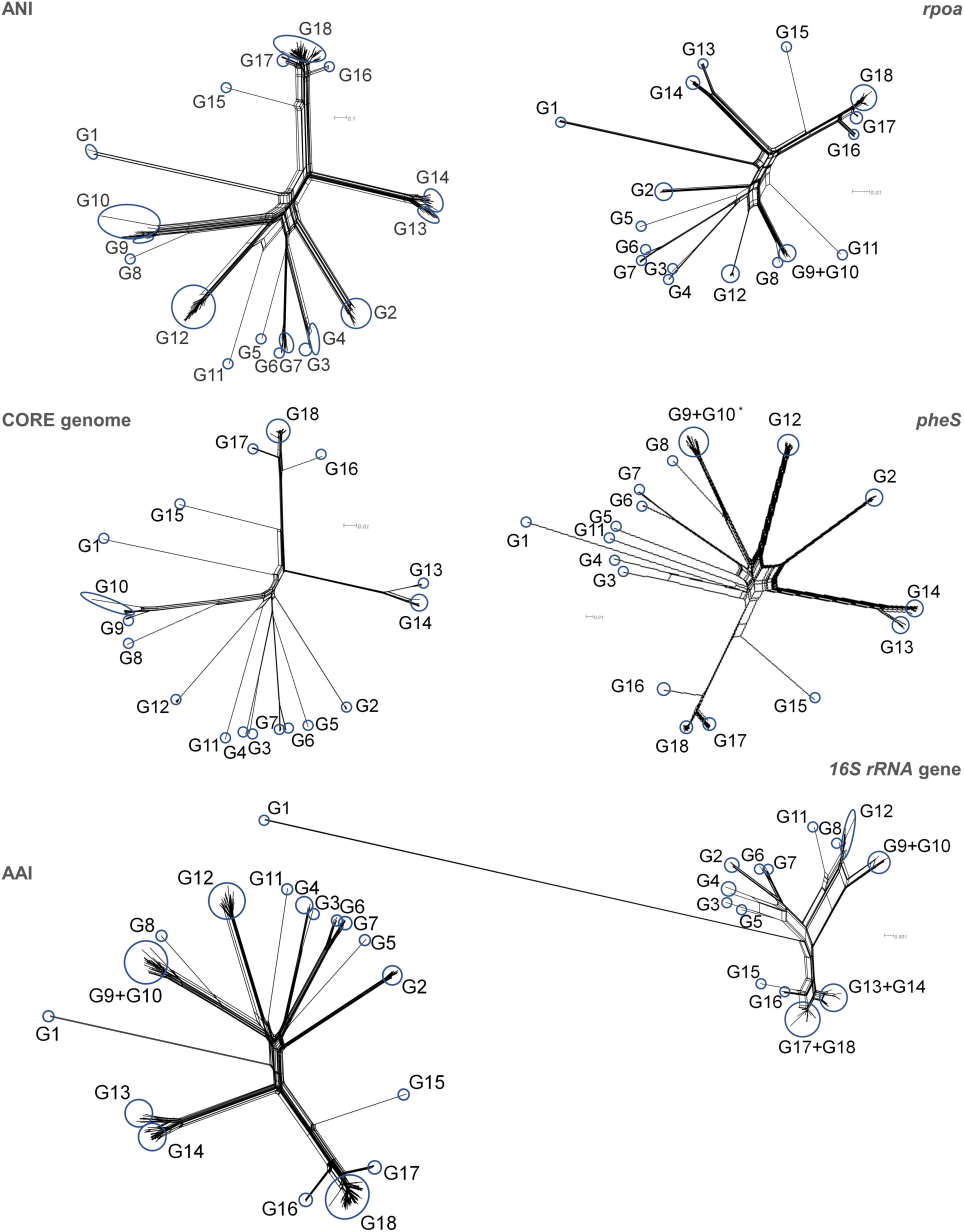
Split decomposition analysis of the phylogenetic trees of ANI, core genome, AAI, *rpoA, pheS*, and 16S rRNA genes. The analysis was performed utilizing gene alignments for the core genome, *rpoA, pheS*, and 16S rRNA genes, and distance matrix for ANI. Blue circles indicate the position of groups on split-decomposed trees. *For clarity of representation, the strain *L. lactis* AV1N that harbored a highly divergent *pheS* sequence, was not included.

**Figure 4:**
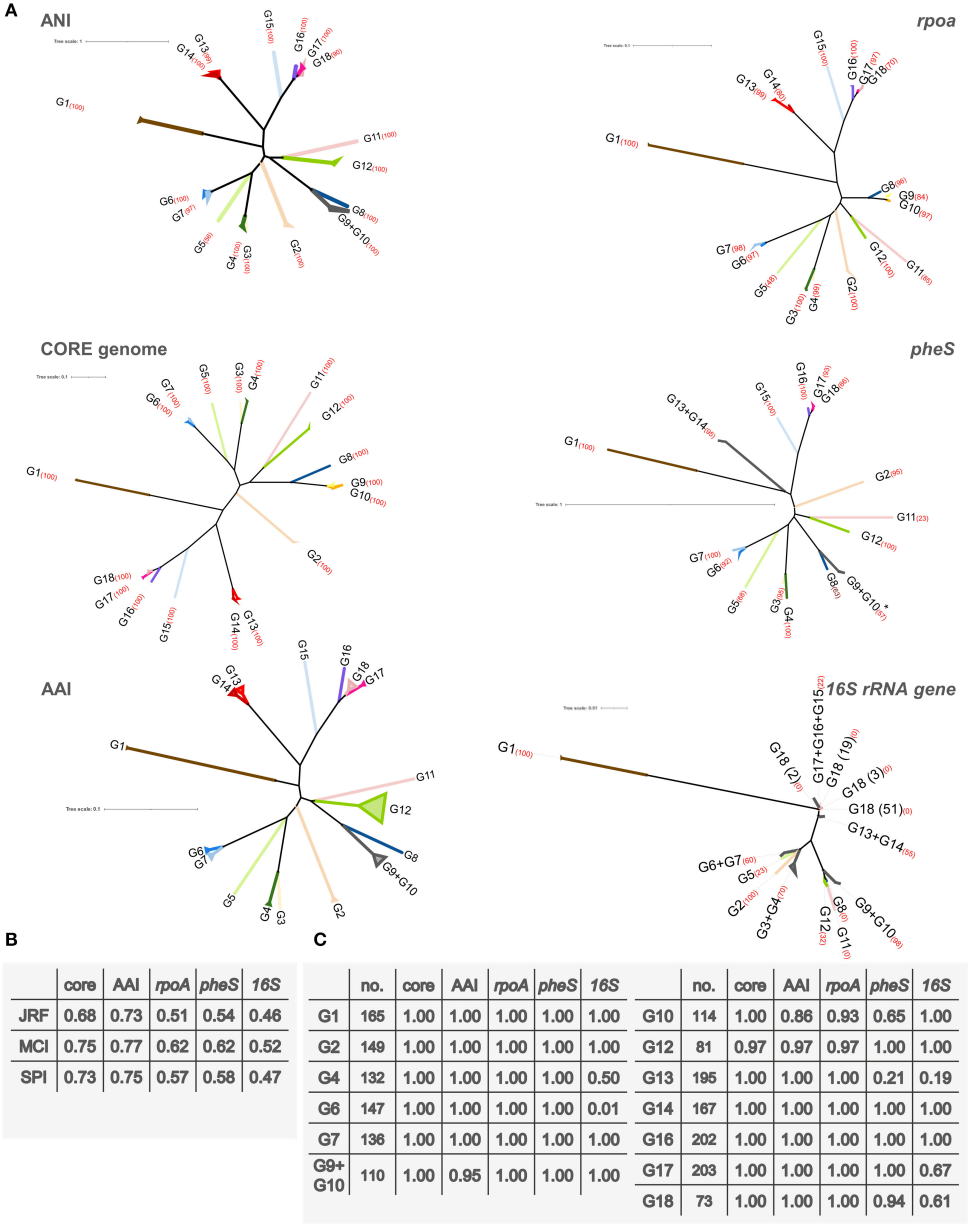
**(A)** Phylogenetic trees representing ANI distances and the alignment of the core genome, *rpoA, pheS*, and the 16S rRNA gene. For core genome, *rpoA, pheS*, and 16S rRNA genes trees, an alignment was produced with Clustal Omega, respectively, and it was used to infer a tree using RAxML. ANI tree was inferred from the ANI distance matrix with R package APE. AAI distance matrix was used to compute the UPGMA tree. Strains are collapsed into their corresponding group. The percentage bootstrap value of each clade is reported in red brackets. *For clarity of representation, the strain *L. lactis* AV1N that harbored a highly divergent *pheS* sequence, was not included. **(B)** Comparison of the ANI tree with the other phylogenetic trees. Normalized similarity score obtained with the generalized Robinson–Foulds metrics Jaccard–Robinson–Foulds (JRF), Mutual Clustering Information (MCI), and Shared Phylogenetic Information (SPI) are reported. **(C)** JRF scores of the 15 groups identified in the ANI tree.

Trees were computed utilizing ANI, the core genome alignment, AAI, and alignment of the genes *rpoA, pheS*, and 16S rRNA (Supplementary Figures 1,2). To evaluate possible conflicting phylogenetic signals, the trees were subjected to split decomposition analysis (Figure 3), which revealed that the core genome alignment yielded the less reticulated network, from which the evolutionary trajectories could be inferred with the lowest ambiguity. ANI, AAI, *rpoA*, and *pheS* yielded more reticulated networks, that corroborated the phylogenetic reconstruction, although with increasing uncertainty in the location of some bifurcations, particularly toward the origin of the main branches. The trees of ANI, AAI, *rpoA*, and *pheS* genes maintained the same general topology with respect to the most peripheral region of the trees, reflecting the most recent evolutionary derivations (Figure 4).

The topology of the ANI phylogram was compared to the other trees, utilizing three “generalized” Robinson–Foulds metrics to establish the consistency of the splits (Figure 4, Supplementary Spreadsheet S3). This analysis indicated that the core genome, AAI, *pheS*, and *rpoA*, in order of decreasing adherence, delineated the same groups identified by ANI analysis. The informative splits corresponding to several groups defined by ANI (i.e., G1, G2, G4, G6, G7, G14, G16, and G17) scored JRF values of 1.00, indicating that the “leaves” of each branch generally coincided in ANI, core genome, AAI, *rpoA*, and *pheS* trees. The phylogeny reconstructed according to ANI, *pheS, rpoA*, the core genome, and AAI were in general agreement in the identification of three major branches: one harboring the groups from G2 to G7, another from G8 to G12, and a third from G13 to G18. The gene *rpoA* was not suitable for phylogenetic positioning of groups G9 and G10, including the strains currently assigned to the species *L. lactis*, whereas the nodes of speciation of the other groups, except for G18 (bootstrap 70%), were identified with very good resolution (bootstraps ≥80%). *pheS* was not able to detect with high confidence (bootstrap <80%) the common speciation of strains assigned to G9, G10, G13, and G18. On the other hand, the 16S rRNA gene presented the lowest phylogenetic signal, generating a highly reticulated network that failed to pinpoint phylogenetic relationships and a maximum likelihood tree where several ANI groups were not resolved. Most of the nodes of the 16S rRNA gene tree presented low bootstrap values, indicating that the confidence of this tree was low.

Phylogenomic relationships closely associated G17 and G18, which were more distantly related to G16, and then to G15 that lay closer to a common ancestor (Figure 2). The nodes from which G15 and G16 descend and separate from G17 and G18 have bootstrap confidence of 100% in the trees of ANI, core genome, *rpoA*, and *pheS*. In turn, G17 and G18 descended from a solid node with a bootstrap of 100% in both ANI and core genome, found also in *rpoA* and *pheS* trees with lower confidence. Coherently, JRF scores of 1.00 indicated that the branches leading to these ANI groups were highly conserved in all these trees. Within G18, a sole strain out of 80 lay on a separate branch, divided from the other 79 strains by a highly solid node (90 and 100% in ANI and the core genome trees, respectively). Most of the strains ascribed to the subspecies *L. mesenteroides* subsp. *cremoris* evolved from a common lineage. On the other side, the strains ascribed to the subspecies *L. mesenteroides* subsp. *mesenteroides* were shuffled within the tree. The clade harboring G13 and G14 was the closest to the one harboring G15, G16, G17, and G18, originating from a node with bootstrap confidence of 100 in the ANI, core genome, and *rpoA* trees. In these trees, G13 and G14 descended from a solid node (99 or 100%) and were highly conserved, presenting a JRF value of 1.00, although the amount of inferred evolutionary change was low. *pheS* failed to separate G13 and G14.

The strictly related species G9 and G10 were resolved in separate branches in the core genome tree by a node with a bootstrap of 100%. Despite pairwise ANI values between the strains of G9 and G10 suggesting their distribution in separate species, the separation was not displayed in the minimum evolution tree of ANI (Figure 2), where the two subgroups of G9 had different locations with respect to G10. In fact, in the ANI tree the subgroup of G9 harboring *L. lactis* KCTC 3773 was separated from G10 by a solid node (bootstrap of 100%), while the other subgroup was placed in a branch of G10 by a more uncertain node (bootstrap of 83%). However, all the trees consistently indicated that G9 and G10 are derived from a lineage that evolved separately also into G8 and that had a common ancestor with G11 and G12. Bootstrap values of 100% corroborated this descendance in both ANI and core genome trees. G11 evolved separately from G12, from which it diverged by a conspicuous evolutionary change.

Another major lineage that could be inferred in the trees of ANI, core genome, AAI, *rpoA*, and *pheS* encompassed G2, G3, G4, G5, G6, and G7. Bootstrap values of 100% in both the ANI and the core genome trees corroborated a pattern of bifurcations that was consistently found in the general traits also in the trees of AAI, *rpoA*, and *pheS*, although with lower bootstrap values. The first bifurcation in the inferred lineage was the branch leading to G2. Subsequently, a branch led to both G3 and G4, reciprocally characterized by a minor amount of inferred evolutionary change. G5 evolved separately from the branch leading to G6 and G7 that, in their turn, were very strictly related. For these groups, the high JRF values reflected the accordance of ANI classification with the core genome, AAI, *rpoA*, and *pheS* trees.

Group G1 was the less closely related species within the genus, presenting on average ANI scores with other groups <82.3%, and being separated from all the other species of *Leuconostoc* by a large amount of inferred evolutionary change. Nonetheless, the placement of *L. fallax* inside the genus was confirmed by AAI, since they presented an AAI value >66% with all the other *Leuconostoc* strains.

## Discussion

Bacterial species can be defined within populations of similar strains on the basis of maximum likelihood methods that determine the point of transition of the evolutionary processes. As a result of common evolution, the strains belonging to the same species share genetic features and thus biological properties. Species delimitations constitute an important challenge in biodiversity studies, mostly for genera such as *Leuconostoc* that have an important role in several industrial and food fermentation processes (Buckenhüskes, 1993; Caplice and Fitzgerald, 1999; Steinkraus, 2002), to produce fermented sausages, fermented vegetables and cereals, and dairy products (e.g., butter, cream, fresh and raw milk, cheeses), and to obtain novel ingredients for cosmeceutical formulations (Ahmadi-Ashtiani et al., 2020).

Taxonomic classifications of the 216 *Leuconostoc* strains based on the core genome sequences indicated that many strains attributed to the same *Leuconostoc* species laying onto paraphyletic branches necessitate reclassification and that the taxonomy of the genus is not entirely resolved. During the revision of the manuscript, a study suggesting the reorganization of *Leuconostoc* taxonomy was published (Kumar et al., 2022). In that manuscript, presenting some overlapping with our survey also in terms of procedures, the proposal of a taxonomic update of the genus *Leuconostoc* was based on ANI and core genome analyses and is consistent with the outcome of our investigation. The phylogenomic reconstruction of the genus herein presented, integrating the information of ANI, core genome, and housekeeping genes complemented the taxonomic delineation with solid information on the phylogenetic lineages evolved within the genus *Leuconostoc*.

In this study, the phylogenomic trees built aligning housekeeping single genes corroborated ANI results. The topology of the phylogenetic trees consistently indicates the remote diversification of most of the species, with a few exceptions. Split decomposition analysis and comparison of the ANI tree with the other ones revealed robust consistency for most of the branches identified, with strong signals for the majority of the nodes identified by ANI, core genome, AAI, *rpoA*, and *pheS* genes, that generally presented very high bootstrap values. 16S rRNA gene yielded weak signals and thus was not suitable to define taxonomy and phylogeny within this genus. According to the consistent results of ANI and phylogenomic trees, the strains currently attributed to *L. mesenteroides* lay onto paraphyletic branches and need to be split into two separate species, corresponding to G16 and G18. Interestingly, these groups are already distinct in GTDB where they are referred to as *Leuconostoc mesenteroides*_B and *Leuconostoc mesenteroides*, respectively, and have been delineated also by Kumar et al. (2022). G18 encompasses most of the strains currently assigned to *L. mesenteroides*, including the type strain, and all the strains belonging to the subspecies *L. mesenteroides* subsp. *cremoris, L. mesenteroides* subsp. *dextranicum, L. mesenteroides* subsp. *jonggajibkimchii*, and *L. mesenteroides* subsp. *mesenteroides*. Nonetheless, the strains belonging to different *L. mesenteroides* subspecies present a genome similarity always >99.1%, higher than the threshold of 98% utilized to delineate subspecies in other bacterial taxa (Minias et al., 2020; Pearce et al., 2021). Accordingly, both in this study and in previous ones the conserved genes failed to differentiate between the subspecies of *L. mesenteroides* (Ricciardi et al., 2020).

G18 is closely related to G17, which includes strains of the species *L. suionicum*, previously recognized as subspecies *L. mesenteroides* subsp. *suionicum* (Jeon et al., 2017). The two strains belonging to G16, currently assigned to *L. mesenteroides*, should form a new species that would require characterization and formal definition (Das et al., 2014; Ramasamy et al., 2014). The strains currently assigned to *L. pseudomesenteroides* should be split into two species, G13 and G14. The former harbors the type strain *L. pseudomesenteroides* NCDO 768^T^, and the latter encompasses also the strains of *L. falkenbergense*, including the type strain *L. falkenbergense* LMG 10779^T^. *L. falkenbergense* has been already recognized as a different species phylogenetically related to *L. pseudomesenteroides* (Wu and Gu, 2021a) and recently added in GTDB.

The strains currently attributed to *L. lactis* should be split into two separate species, corresponding to the groups G9 and G10. Consistently, in GTDB the groups are already distinct and referred to as *Leuconostoc lactis*_A and *Leuconostoc lactis*. G10 encompasses the type strain *L. lactis* JCM 6123^T^, the strain KFRI01 named *L. garlicum*, a species that is not formally recognized, and *L. citreum* 1300_LCIT. G9 harbors *L. lactis* KCTC 3773, previously identified as the type strain of *L. argentinum*, species that is not anymore accepted (Dicks et al., 1993; Vancanneyt et al., 2006). Within both G10 and G9, subgroups of strains putatively ascribable to different subspecies can be identified, that present ANI scores between each other lower than 98%. Therefore, further phenotypic and genotypic studies could investigate whether subspecies have to be created within G10 and G9 groups. The strains of *L. gelidum* and *L. gasicomitatum* fall into G6 and G7, respectively, which include the type strains *L. gelidum* subsp. *gelidum* KCTC 3527^T^ and *L. gasicomitatum* LMG_18811^T^. This classification confirms the observations by Wu and Gu (2021b) that rejected the proposal to recognize *L. gasicomitatum* as a subspecies of *L. gelidum* (*L. gelidum* subsp. *gasicomitatum*) (Rahkila et al., 2014). Moreover, G7 includes also the type strain *L. inhae* KCTC 3774^T^, suggesting that *L. inhae* and *L. gasicomitatum* could belong to the same species. No clear correlation could be established between the phylogenetic relationships and the source of isolation of the strains, except for G2 (*L. carnosum)* and G14 (*L. falkenbergense*) that encompassed mostly strains isolated from meat and dairy products, respectively (Supplementary Table S1) (Raimondi et al., 2018; Wu and Gu, 2021a). The evolutive force that shaped the speciation of *Leuconostoc* deserves deeper comparative genomics and functional analysis.

The results of this study confirmed and deepened the evidence on the evolutionary relationship among *Leuconostoc* species and may provide a basis for a possible future reorganization of the genus, as summarized in Supplementary Table S1. However, any update in the organization of *Leuconostoc* genus, such as the creation of new species and/or subspecies, would require, all the biochemical and physiological data based on strains survey that support the creation of new species and the reclassification of others.

## Data availability statement

The original contributions presented in the study are included in the article/Supplementary material, further inquiries can be directed to the corresponding author/s.

## Author contributions

Conceptualization: SR, AA, and MR. Investigation: SR, FC, and GS. Data curation: FC, AA, SV, and SC. Formal Analysis: AA. Visualization: SR, FC, and AA. Writing—original draft: All authors. Funding acquisition: MR and SV. Supervision: MR. All authors contributed to the article and approved the submitted version.

## Conflict of interest

The authors declare that the research was conducted in the absence of any commercial or financial relationships that could be construed as a potential conflict of interest.

## Publisher's note

All claims expressed in this article are solely those of the authors and do not necessarily represent those of their affiliated organizations, or those of the publisher, the editors and the reviewers. Any product that may be evaluated in this article, or claim that may be made by its manufacturer, is not guaranteed or endorsed by the publisher.
