# Supplementary material for: Phylogenomic analysis of the genus Leuconostoc
Source: Front Microbiol. 2022 Jul 25;13:897656. doi: 10.3389/fmicb.2022.897656 (PMC9358442; doi:10.3389/fmicb.2022.897656)
Supplement: Supplementary file 4 [file Data_Sheet_1.PDF]

Phylogenomic analysis of the genus *Leuconostoc*

Stefano Raimondi[1,†], Francesco Candeliere[1,†], Alberto Amaretti[1,2], Stefania Costa[3], Silvia Vertuani[4], Gloria Spampinato[1], Maddalena Rossi[1,2,*]

[1]Department of Life Sciences, University of Modena and Reggio Emilia, Modena, Italy

[2]Biogest Siteia, University of Modena and Reggio Emilia, Reggio Emilia, Italy

[3]Department of Chemical, Pharmaceutical and Agricultural Sciences – DOCPAS, University of Ferrara, Ferrara, Italy

[4]Department of Life Sciences and Biotechnology, University of Ferrara, Ferrara, Italy

† These authors equally contributed to the work

*** Correspondence:**
Corresponding Author
maddalena.rossi@unimore.it

Supplementary Material

Supplementary Table 1. List of the 221 *Leuconostoc* strains corresponding to the available genome assemblies on NCBI database in September 2021. The assignment to groups according to the ANI tree is reported. (N/A, not available). * Name changes according to the evidence provided in this study and in Kumar et al. (2022).

Group	Species	Subspecies	Strain	Type strain	Size (Mb)	GC%	Assembly	Isolation source	Completeness (%)	Contamination (%)	16S rRNA gene Genbank accession n°	16S rRNA seq. identity (%)	GTDB species cluster	Putative name *
**G1**	***L. fallax***		**ATCC_700006**	T	1.7	37.7	GCA_004354645.1	Vegetable/Fruit/Corn/Plant	99.3	0.5	AF360738	100.0	s__Leuconostoc fallax	
**G1**	*L. fallax*		KCTC_3537		1.6	37.5	GCA_000165675.2	Vegetable/Fruit/Corn/Plant	95.7	0.5			s__Leuconostoc fallax	
**G2**	*L. carnosum*		WC0322		1.9	37.0	GCA_008690865.1	Meat	99.5	1.1			s__Leuconostoc carnosum	
**G2**	*L. carnosum*		WC0323		1.8	37.2	GCA_008690845.1	Meat	99.5	1.1			s__Leuconostoc carnosum	
**G2**	*L. carnosum*		WC0324		1.8	37.1	GCA_008690825.1	Meat	99.5	1.1			s__Leuconostoc carnosum	
**G2**	*L. carnosum*		WC0328		1.8	37.1	GCA_008689695.1	Meat	99.0	1.1			s__Leuconostoc carnosum	
**G2**	*L. carnosum*		WC0319		1.7	37.1	GCA_008690925.1	Meat	99.5	1.1			s__Leuconostoc carnosum	
**G2**	*L. carnosum*		JB16_JB16		1.8	37.1	GCA_000300135.1	Vegetable/Fruit/Corn/Plant	99.5	1.1			s__Leuconostoc carnosum	
**G2**	***L. carnosum***		**DSM_5576**	T	1.8	37.0	GCA_014207505.1	Meat	99.5	1.1	NR_040811	99.9	s__Leuconostoc carnosum	
**G2**	*L. carnosum*		WC0318		1.8	37.2	GCA_008690985.1	Meat	99.5	1.1			s__Leuconostoc carnosum	
**G2**	*L. carnosum*		MFPC16A2803		1.8	37.0	GCA_900322415.1	Meat	99.5	1.1			s__Leuconostoc carnosum	
**G2**	*L. carnosum*		WC0325		1.8	37.2	GCA_008690785.1	Meat	99.5	1.1			s__Leuconostoc carnosum	
**G2**	*L. carnosum*		WC0327		1.8	37.2	GCA_008689755.1	Meat	99.5	1.1			s__Leuconostoc carnosum	
**G2**	*L. carnosum*		WC0326		1.8	37.2	GCA_008689765.1	Meat	99.5	1.1			s__Leuconostoc carnosum	
**G2**	*L. carnosum*		WC0321		1.8	37.1	GCA_008690875.1	Meat	99.5	1.1			s__Leuconostoc carnosum	
**G2**	*L. carnosum*		MFPA29A1405		1.7	37.3	GCA_900322425.1	Meat	99.5	1.1			s__Leuconostoc carnosum	
**G2**	*L. carnosum*		CBA3620		1.7	37.3	GCA_007954525.1	Vegetable/Fruit/Corn/Plant	99.5	1.1			s__Leuconostoc carnosum	
**G2**	*L. carnosum*		WC0320		1.8	37.1	GCA_008690965.1	Meat	99.5	1.1			s__Leuconostoc carnosum	
**G2**	*L. carnosum*		WC0329		1.7	37.2	GCA_008689675.1	Meat	99.5	1.1			s__Leuconostoc carnosum	
**G3**	***L. rapi***		**DSM 27776**	T	1.9	38.0	GCF_016908715.1	-	100.0	1.2	NR_136799	100.0	N/A	
**G4**	***L. kimchii***		**IMSNU_11154**	T	2.1	37.9	GCA_000092505.1	Vegetable/Fruit/Corn/Plant	100.0	0.0	NR_075014	100.0	s__Leuconostoc kimchii	
**G4**	*Leuconostoc sp.*		C2		1.9	37.9	GCA_000219785.1	Vegetable/Fruit/Corn/Plant	100.0	0.0			s__Leuconostoc kimchii	*L. kimchii*
**G4**	*L. kimchii*		NKJ218		2.0	37.8	GCA_004551615.1	Vegetable/Fruit/Corn/Plant	100.0	0.0			s__Leuconostoc kimchii	
**G5**	***L. miyukkimchii***		**JCM 17445**	T	1.9	36.3	GCF_019656055.1	Brown algae kimchi	99.4	1.0	NR_109072	99.9	N/A	
**G6**	*L. gelidum*	*gelidum*	TMW_2.1618		1.8	36.9	GCA_006770305.1	Meat	99.4	0.2			s__Leuconostoc gelidum	
**G6**	***L. gelidum***	***gelidum***	**KCTC_3527**	T	2.0	36.6	GCA_000166715.2	Vegetable/Fruit/Corn/Plant	99.4	0.2	AF175402	99.8	s__Leuconostoc gelidum	
**G6**	*L. gelidum*		JB7		1.9	36.7	GCA_000298875.1	Vegetable/Fruit/Corn/Plant	99.4	0.2			s__Leuconostoc gelidum	
**G7**	*L. inhae*		KSL4-2		2.1	36.6	GCA_900016165.1	Food	99.3	0.1			s__Leuconostoc inhae	*L. inhae / L. gasicomitatum*
**G7**	*L. inhae*		C120c		2.0	36.5	GCA_900009505.1	Vegetable/Fruit/Corn/Plant	99.4	0.0			s__Leuconostoc inhae	*L. inhae / L. gasicomitatum*
**G7**	*L. gelidum*	*gasicomitatum*	CBA3613		1.8	36.9	GCA_013403485.1	-	100.0	0.1			s__Leuconostoc inhae	*L. inhae / L. gasicomitatum*
**G7**	***L. gelidum***	***gasicomitatum***	**LMG_18811**	T	2.0	36.7	GCA_000196855.1	Meat	99.4	0.0	NR_074997	100.0	s__Leuconostoc inhae	*L. inhae / L. gasicomitatum*
**G7**	*L. gelidum*	*gasicomitatum*	MFPA44A1401		1.9	36.8	GCA_900218135.1	Meat	100.0	0.0			s__Leuconostoc inhae	*L. inhae / L. gasicomitatum*
**G7**	***L. inhae***		**KCTC_3774**	T	2.3	36.4	GCA_000166735.2	Vegetable/Fruit/Corn/Plant	95.8	6.1	NR_025204	99.8	s__Leuconostoc inhae	*L. inhae / L. gasicomitatum*
**G7**	*L. gelidum*	*gasicomitatum*	TMW_2.1619		1.9	36.9	GCA_009296125.1	Meat	99.6	0.6			s__Leuconostoc inhae	*L. inhae / L. gasicomitatum*
**G7**	*L. gelidum*	*gasicomitatum*	PB1e		2.1	36.5	GCA_900016175.1	Vegetable/Fruit/Corn/Plant	99.4	0.0			s__Leuconostoc inhae	*L. inhae / L. gasicomitatum*
**G7**	*L. gelidum*	*gasicomitatum*	LEKG1		2.1	36.9	GCA_001536305.1	Vegetable/Fruit/Corn/Plant	100.0	0.1			s__Leuconostoc inhae	*L. inhae / L. gasicomitatum*

Group	Species	Subspecies	Strain	Type strain	Size (Mb)	GC%	Assembly	Isolation source	Completeness (%)	Contamination (%)	16S rRNA gene Genbank accession n°	16S rRNA seq. identity (%)	GTDB species cluster	Putative name *
**G7**	*L. inhae*		PB1a		2.1	36.6	GCA_900016185.1	Vegetable/Fruit/Corn/Plant	99.4	0.6			s__Leuconostoc inhae	*L. inhae / L. gasicomitatum*
**G7**	*L. inhae*		PL111		2.1	36.5	GCA_900016205.1	Vegetable/Fruit/Corn/Plant	99.4	0.0			s__Leuconostoc inhae	*L. inhae / L. gasicomitatum*
**G7**	*L. gelidum*	*gasicomitatum*	C122c		2.0	36.6	GCA_900016145.1	Vegetable/Fruit/Corn/Plant	98.9	0.2			s__Leuconostoc inhae	*L. inhae / L. gasicomitatum*
**G8**	***L. holzapfelii***		**CCUG_54536**	T	1.8	44.0	GCA_012396485.1	Vegetable/Fruit/Corn/Plant	99.4	0.3	AM600682	99.9	s__Leuconostoc holzapfelii	
**G9**	*L. lactis (argentinum)*		KCTC_3773		1.7	42.9	GCA_000179875.1	Dairy	99.3	0.0	AF175403	99.9	s__Leuconostoc lactis_A	putative sp. nov. G9
**G9**	*L. lactis*		1001095IJ_161003_G05		1.7	43.3	GCA_015553465.1	Human sample	99.8	0.0			N/A	putative sp. nov. G9
**G9**	*L. lactis*		LN24		1.7	42.9	GCA_002092695.1	Dairy	99.3	0.0			s__Leuconostoc lactis_A	putative sp. nov. G9
**G9**	*L. lactis*		LN19		1.7	42.9	GCA_002092595.1	Dairy	99.3	0.0			s__Leuconostoc lactis_A	putative sp. nov. G9
**G9**	*L. lactis*		CBA3622		1.8	42.9	GCA_007954625.1	Vegetable/Fruit/Corn/Plant	99.8	0.0			s__Leuconostoc lactis_A	putative sp. nov. G9
**G9**	*L. lactis*		WIKIM21		1.8	43.1	GCA_001411775.1	Vegetable/Fruit/Corn/Plant	99.8	0.0			s__Leuconostoc lactis_A	putative sp. nov. G9
**G10**	*L. lactis*		KCTC_3528		2.0	42.6	GCA_000185085.2	-	89.6	17.8			Undefined (Failed Quality Check)	
**G10**	*L. lactis*		AV1n		1.7	43.2	GCA_009795665.1	Vegetable/Fruit/Corn/Plant	85.7	0.0			s__Leuconostoc lactis	
**G10**	*L. lactis*		CBA3626		1.8	43.1	GCA_007954665.1	Vegetable/Fruit/Corn/Plant	99.8	0.5			s__Leuconostoc lactis	
**G10**	*L. lactis*		WiKim40		1.8	43.1	GCA_001698145.1	Vegetable/Fruit/Corn/Plant	99.8	0.0			s__Leuconostoc lactis	
**G10**	*L. lactis*		SBC001		1.8	43.1	GCA_014050705.1	Vegetable/Fruit/Corn/Plant	99.8	0.0			s__Leuconostoc lactis	
**G10**	*L. lactis*		KACC_91922		1.7	43.4	GCA_000709265.1	Vegetable/Fruit/Corn/Plant	99.8	0.0			s__Leuconostoc lactis	
**G10**	***L. lactis***		**JCM_6123**	T	1.7	43.3	GCA_014651235.1	Collection strain	99.8	0.0	NR_040823	100.0	s__Leuconostoc lactis	
**G10**	*L. lactis*		aa_143		1.7	43.2	GCA_004167235.1	Human sample	99.8	0.0			s__Leuconostoc lactis	
**G10**	*L. lactis*		NBRC_12455		1.6	43.5	GCA_006539105.1	Collection strain	99.8	0.0			s__Leuconostoc lactis	
**G10**	*L. lactis*		CCK940		1.7	43.3	GCA_002287365.1	Vegetable/Fruit/Corn/Plant	99.8	0.0			s__Leuconostoc lactis	
**G10**	*L. citreum*		1300_LCIT		1.8	43.1	GCA_001062635.1	Human sample	99.8	0.0			s__Leuconostoc lactis	
**G10**	*L. lactis*		1001262B_160229_C9		1.7	43.6	GCA_015551285.1	Human sample	99.8	0.0			N/A	
**G10**	*L. garlicum*		KFRI01		1.7	43.3	GCA_001998805.1	Vegetable/Fruit/Corn/Plant	99.8	0.0			s__Leuconostoc lactis	
**G10**	*L. lactis*		BIOML-A1		1.8	43.1	GCA_009678855.1	Human sample	99.8	0.0			s__Leuconostoc lactis	
**G10**	*L. lactis*		CBA3625		1.8	43.3	GCA_007954605.1	Vegetable/Fruit/Corn/Plant	99.8	0.0			s__Leuconostoc lactis	
**G11**	***L. palmae***		**JCM 16944**	T	1.4	35.4	GCF_019656075.1	Palm wine	99.3	0.6	NR_042695	99.9	N/A	
**G12**	*L. citreum*		DmW_111		1.8	38.8	GCA_002115685.1	Insect	99.3	0.8			s__Leuconostoc citreum	
**G12**	*L. citreum*		LBAE_C10		1.9	38.8	GCA_000239895.2	Vegetable/Fruit/Corn/Plant	99.9	0.1			s__Leuconostoc citreum	
**G12**	*L. citreum*		CBA3627		1.9	38.9	GCA_008033195.1	Vegetable/Fruit/Corn/Plant	99.9	0.6			s__Leuconostoc citreum	
**G12**	*L. citreum*		CBA3621		1.9	38.9	GCA_007954785.1	Vegetable/Fruit/Corn/Plant	99.9	0.6			s__Leuconostoc citreum	
**G12**	*L. citreum*		G04		1.8	38.8	GCA_006381855.1	Vegetable/Fruit/Corn/Plant	99.9	0.0			s__Leuconostoc citreum	
**G12**	*L. citreum*		37		2.0	38.9	GCA_015277835.1	Dairy	99.9	0.0			N/A	
**G12**	*L. citreum*		1301_LGAS		1.8	39.0	GCA_001077275.1	Human sample	99.9	0.2			s__Leuconostoc citreum	
**G12**	*L. citreum*		NBRC_102476		1.8	38.9	GCA_007989545.1	Collection strain	99.9	0.0			s__Leuconostoc citreum	
**G12**	*L. citreum*		DmW_137		1.9	38.9	GCA_014927285.1	Insect	99.9	0.0			N/A	
**G12**	*L. citreum*		F192-5		2.1	38.4	GCA_008326505.1	Vegetable/Fruit/Corn/Plant	99.9	0.0			s__Leuconostoc citreum	
**G12**	*L. citreum*		CW28		2.0	38.7	GCA_002591805.1	Vegetable/Fruit/Corn/Plant	99.3	0.0			s__Leuconostoc citreum	
**G12**	***L. citreum***		**ATCC_49370**	T	1.8	39.0	GCA_004354555.1	Collection strain	99.9	0.0	NR_041727	99.8	s__Leuconostoc citreum	
**G12**	*L. citreum*		NRIC_1776		1.8	38.9	GCA_008326545.1	Vegetable/Fruit/Corn/Plant	99.9	0.0			s__Leuconostoc citreum	
**G12**	*L. citreum*		WiKim0096		1.8	39.0	GCA_016406245.1	Vegetable/Fruit/Corn/Plant	99.7	0.0			N/A	
**G12**	*L. citreum*		CBA3624		1.9	39.0	GCA_007954565.1	Vegetable/Fruit/Corn/Plant	99.9	0.6			s__Leuconostoc citreum	
**G12**	*L. citreum*		EFEL_2700		1.9	39.0	GCA_002804045.1	Vegetable/Fruit/Corn/Plant	99.3	0.0			s__Leuconostoc citreum	
**G12**	*L. citreum*		TR116		1.8	38.8	GCA_004359915.1	Vegetable/Fruit/Corn/Plant	99.9	0.0			s__Leuconostoc citreum	
**G12**	*L. citreum*		TMW21194		1.7	38.9	GCA_009792915.1	Food	99.9	0.0			s__Leuconostoc citreum	

Group	Species	Subspecies	Strain	Type strain	Size (Mb)	GC%	Assembly	Isolation source	Completeness (%)	Contamination (%)	16S rRNA gene Genbank accession n°	16S rRNA seq. identity (%)	GTDB species cluster	Putative name *
**G12**	*L. citreum*		WiKim0101		2.0	38.9	GCA_009707745.1	Vegetable/Fruit/Corn/Plant	99.2	0.2			s__Leuconostoc citreum	
**G12**	*Leuconostoc sp.*		LN180020		1.9	39.0	GCA_014844115.1	Vegetable/Fruit/Corn/Plant	99.9	0.0			N/A	*L. citreum*
**G12**	*L. citreum*		CBA3623		2.0	38.9	GCA_007954705.1	Vegetable/Fruit/Corn/Plant	99.3	0.0			s__Leuconostoc citreum	
**G12**	*L. citreum*		NRRL_B-1299		1.8	39.0	GCA_000820985.2	-	99.9	0.0			s__Leuconostoc citreum	
**G12**	*L. citreum*		TR153		1.9	38.7	GCA_006406175.1	Vegetable/Fruit/Corn/Plant	99.9	0.0			s__Leuconostoc citreum	
**G12**	*L. citreum*		SG0255		1.9	38.8	GCA_014060965.1	Vegetable/Fruit/Corn/Plant	99.9	0.1			s__Leuconostoc citreum	
**G12**	*L. citreum*		LBAE_C11		2.0	38.7	GCA_000239915.2	Vegetable/Fruit/Corn/Plant	99.9	0.0			s__Leuconostoc citreum	
**G12**	*L. citreum*		LBAE_E16		1.8	38.9	GCA_000239935.2	Vegetable/Fruit/Corn/Plant	99.9	0.0			s__Leuconostoc citreum	
**G12**	*L. citreum*		BD1707		1.8	38.9	GCA_013432155.1	Dairy	99.9	0.0			s__Leuconostoc citreum	
**G12**	*L. citreum*		NRRL_B-742		1.7	39.2	GCA_000820965.2	-	99.3	0.0			s__Leuconostoc citreum	
**G12**	*L. citreum*		KM20		1.9	38.9	GCA_000026405.1	Vegetable/Fruit/Corn/Plant	99.9	0.0			s__Leuconostoc citreum	
**G13**	*L. pseudomesenteroides*		LMG_11483		2.0	39.0	GCA_014634765.1	-	99.3	0.0			s__Leuconostoc pseudomesenteroides	
**G13**	*L. pseudomesenteroides*		LMG_11482		2.1	38.9	GCA_014634745.1	Vegetable/Fruit/Corn/Plant	99.8	0.0			s__Leuconostoc pseudomesenteroides	
**G13**	***L. pseudomesenteroides***		**NCDO_768**	T	2.1	38.9	GCA_012396745.1	Vegetable/Fruit/Corn/Plant	99.8	0.0	NR_040814	99.6	s__Leuconostoc pseudomesenteroides	
**G13**	*L. pseudomesenteroides*		FDAARGOS_1003		2.1	39.0	GCA_016127255.1	Vegetable/Fruit/Corn/Plant	99.8	0.0			N/A	
**G13**	*Leuconostoc sp.*		S51		2.3	38.8	GCA_016517085.1	Insect	99.8	0.0			N/A	*L. pseudomesenteroides*
**G13**	*L. pseudomesenteroides*		17_2		2.3	38.5	GCA_014634725.1	Vegetable/Fruit/Corn/Plant	99.8	0.0			s__Leuconostoc pseudomesenteroides	
**G13**	*L. pseudomesenteroides*		CBA3630		2.3	39.0	GCA_008033175.1	Vegetable/Fruit/Corn/Plant	99.8	0.2			s__Leuconostoc pseudomesenteroides	
**G13**	*L. pseudomesenteroides*		TR070		2.2	38.8	GCA_006382035.1	Vegetable/Fruit/Corn/Plant	99.8	0.0			s__Leuconostoc pseudomesenteroides	
**G13**	*L. pseudomesenteroides*		KCTC_3652		3.2	38.3	GCA_000185065.2	Vegetable/Fruit/Corn/Plant	99.1	46.4			Undefined (Failed Quality Check)	
**G14**	*L. falkenbergense*		LMG_18969		1.9	39.0	GCA_014634865.1	Vegetable/Fruit/Corn/Plant	99.8	0.0			s__Leuconostoc pseudomesenteroides_B	
**G14**	*L. pseudomesenteroides*		LMGTW8		2.0	39.0	GCA_002092295.1	Dairy	100.0	0.0			s__Leuconostoc pseudomesenteroides_B	*L. falkenbergense*
**G14**	*L. pseudomesenteroides*		1159		2.0	39.0	GCA_000686465.1	Dairy	100.0	0.0			s__Leuconostoc pseudomesenteroides_B	*L. falkenbergense*
**G14**	*L. pseudomesenteroides*		PS12		1.9	39.1	GCA_000686505.1	Dairy	100.0	0.0			s__Leuconostoc pseudomesenteroides_B	*L. falkenbergense*
**G14**	*L. pseudomesenteroides*		LMGTW1		2.0	39.0	GCA_002092235.1	Dairy	100.0	0.0			s__Leuconostoc pseudomesenteroides_B	*L. falkenbergense*
**G14**	*Leuconostoc sp.*		BM2		2.1	39.0	GCA_002095565.1	Dairy	100.0	0.0			s__Leuconostoc pseudomesenteroides_B	*L. falkenbergense*
**G14**	*L. pseudomesenteroides*		FDAARGOS_1004		2.1	39.1	GCA_016127035.1	-	100.0	0.0			N/A	*L. falkenbergense*
**G14**	*L. pseudomesenteroides*		LMGH97		2.0	39.0	GCA_002072585.1	Dairy	100.0	0.0			s__Leuconostoc pseudomesenteroides_B	*L. falkenbergense*
**G14**	*L. pseudomesenteroides*		BM2		2.0	39.0	GCA_002092535.1	Dairy	100.0	0.0			s__Leuconostoc pseudomesenteroides_B	*L. falkenbergense*
**G14**	*L. pseudomesenteroides*		LMGH280		2.0	39.1	GCA_002072555.1	Dairy	100.0	0.7			s__Leuconostoc pseudomesenteroides_B	*L. falkenbergense*
**G14**	*L. pseudomesenteroides*		LMGH95		2.0	39.0	GCA_002072565.1	Dairy	100.0	0.2			s__Leuconostoc pseudomesenteroides_B	*L. falkenbergense*
**G14**	*L. pseudomesenteroides*		LMGH83		2.1	39.1	GCA_002072475.1	Dairy	98.4	1.4			s__Leuconostoc pseudomesenteroides_B	*L. falkenbergense*
**G14**	*L. pseudomesenteroides*		LMGH284		2.1	39.0	GCA_002072575.1	Dairy	99.2	2.0			s__Leuconostoc pseudomesenteroides_B	*L. falkenbergense*
**G14**	*L. pseudomesenteroides*		LMGCF15		1.9	39.1	GCA_002092375.1	Dairy	100.0	0.0			s__Leuconostoc pseudomesenteroides_B	*L. falkenbergense*
**G14**	*L. pseudomesenteroides*		LMGH278		1.9	39.1	GCA_002072495.1	Dairy	100.0	0.0			s__Leuconostoc pseudomesenteroides_B	*L. falkenbergense*
**G14**	*L. falkenbergense*		C		2.3	38.6	GCA_014634785.1	Dairy	100.0	0.0			s__Leuconostoc pseudomesenteroides_B	
**G14**	*L. pseudomesenteroides*		KMB_610		2.0	39.0	GCA_003346375.1	Dairy	100.0	0.0			s__Leuconostoc pseudomesenteroides_B	*L. falkenbergense*
**G14**	*L. pseudomesenteroides*		4882		2.0	39.1	GCA_000297375.1	Dairy	93.2	0.2			s__Leuconostoc pseudomesenteroides_B	*L. falkenbergense*
**G14**	*L. pseudomesenteroides*		LMGTW3		1.9	39.1	GCA_002092355.1	Dairy	100.0	0.0			s__Leuconostoc pseudomesenteroides_B	*L. falkenbergense*
**G14**	*L. pseudomesenteroides*		LMGH61		2.0	39.1	GCA_002072515.1	Dairy	100.0	0.0			s__Leuconostoc pseudomesenteroides_B	*L. falkenbergense*
**G14**	*L. pseudomesenteroides*		LN23		2.0	39.1	GCA_002092645.1	Dairy	100.0	0.2			s__Leuconostoc pseudomesenteroides_B	*L. falkenbergense*
**G14**	***L. falkenbergense***		**LMG_10779**	T	2.0	39.1	GCA_014634805.1	Collection strain	100.0	0.2	HM443956	100.0	s__Leuconostoc pseudomesenteroides_B	
**G14**	*L. pseudomesenteroides*		LMGTW6		1.9	39.1	GCA_002092255.1	Dairy	100.0	0.0			s__Leuconostoc pseudomesenteroides_B	*L. falkenbergense*

Group	Species	Subspecies	Strain	Type strain	Size (Mb)	GC%	Assembly	Isolation source	Completeness (%)	Contamination (%)	16S rRNA gene Genbank accession n°	16S rRNA seq. identity (%)	GTDB species cluster	Putative name *
**G14**	*L. pseudomesenteroides*		AMBR10		2.3	39.2	GCA_901830415.1	Human sample	100.0	0.7			s__Leuconostoc pseudomesenteroides_B	*L. falkenbergense*
**G14**	*L. pseudomesenteroides*		LMGH100		2.1	39.1	GCA_002072505.1	Dairy	98.7	2.6			s__Leuconostoc pseudomesenteroides_B	*L. falkenbergense*
**G14**	*L. pseudomesenteroides*		HPK01		2.0	39.1	GCA_002092075.1	Dairy	100.0	0.0			s__Leuconostoc pseudomesenteroides_B	*L. falkenbergense*
**G14**	*L. pseudomesenteroides*		LMGCF06		2.0	39.1	GCA_002092035.1	Dairy	100.0	0.0			s__Leuconostoc pseudomesenteroides_B	*L. falkenbergense*
**G14**	*L. pseudomesenteroides*		LN02		1.9	39.1	GCA_002092555.1	Dairy	100.0	0.0			s__Leuconostoc pseudomesenteroides_B	*L. falkenbergense*
**G14**	*L. pseudomesenteroides*		LN12		1.9	39.1	GCA_002092635.1	Dairy	100.0	0.0			s__Leuconostoc pseudomesenteroides_B	*L. falkenbergense*
**G14**	*L. pseudomesenteroides*		LMGCF08		2.2	39.1	GCA_002092165.1	Dairy	100.0	11.1			Undefined (Failed Quality Check)	*L. falkenbergense*
**G15**	***L. litchii***		**MB17**	T	1.9	35.8	GCA_008107645.1	Vegetable/Fruit/Corn/Plant	100.0	0.5	LC259518	99.5	s__Leuconostoc litchii	
**G16**	*L. mesenteroides*	*mesenteroides*	CBA3607		1.9	37.0	GCA_009676745.1	Vegetable/Fruit/Corn/Plant	100.0	0.0			s__Leuconostoc mesenteroides_B	putative sp. nov. G16
**G16**	*L. mesenteroides*		CBA3628		1.9	37.0	GCA_007954745.1	Vegetable/Fruit/Corn/Plant	100.0	0.0			s__Leuconostoc mesenteroides_B	putative sp. nov. G16
**G17**	*L. suionicum*		CECT_8484		2.1	37.3	GCA_900289205.1	Vegetable/Fruit/Corn/Plant	100.0	0.5			s__Leuconostoc suionicum	
**G17**	*L. suionicum*		CECT_9216		2.1	37.3	GCA_900290145.1	Vegetable/Fruit/Corn/Plant	100.0	0.5			s__Leuconostoc suionicum	
**G17**	*L. suionicum*		CECT_8486		2.1	37.3	GCA_900289255.1	Vegetable/Fruit/Corn/Plant	100.0	0.5			s__Leuconostoc suionicum	
**G17**	*L. suionicum*		DmW_98		2.1	37.3	GCA_014927295.1	Insect	100.0	0.0			N/A	
**G17**	*L. suionicum*		LT-38		2.0	37.6	GCA_002370395.1	Collection strain	100.0	0.0			s__Leuconostoc suionicum	
**G17**	***L. suionicum***		**DSM_20241**	T	2.1	37.6	GCA_001891125.1	-	100.0	0.0	NR_109003	100.0	s__Leuconostoc suionicum	
**G18**	*L. mesenteroides*		AtHG050		2.0	37.7	GCA_004368765.1	Vegetable/Fruit/Corn/Plant	100.0	1.1			s__Leuconostoc mesenteroides	
**G18**	*L. mesenteroides*		CECT_9268		2.1	37.7	GCA_900290155.1	Vegetable/Fruit/Corn/Plant	99.8	0.5			s__Leuconostoc mesenteroides	
**G18**	*L. mesenteroides*		CECT_9266		2.1	37.7	GCA_900312985.1	Vegetable/Fruit/Corn/Plant	99.8	0.5			s__Leuconostoc mesenteroides	
**G18**	*L. mesenteroides*	*mesenteroides*	KMB_608		2.0	37.7	GCA_003346165.1	Dairy	100.0	0.5			s__Leuconostoc mesenteroides	
**G18**	*L. mesenteroides*	*mesenteroides*	LN32		1.9	37.8	GCA_002092705.1	Dairy	100.0	0.5			s__Leuconostoc mesenteroides	
**G18**	*L. mesenteroides*		GL1		1.8	38.1	GCA_001541125.1	Dairy	100.0	1.3			s__Leuconostoc mesenteroides	
**G18**	*L. mesenteroides*	*mesenteroides*	KMB_611		2.1	37.5	GCA_003346345.1	Dairy	100.0	1.1			s__Leuconostoc mesenteroides	
**G18**	*L. mesenteroides*	*dextranicum*	NBRC_100495		1.7	37.9	GCA_007990145.1	Collection strain	100.0	0.5			s__Leuconostoc mesenteroides	
**G18**	*L. mesenteroides*		NCTC12954		2.1	37.7	GCA_900461615.1	Collection strain	100.0	1.1			s__Leuconostoc mesenteroides	
**G18**	*L. mesenteroides*		LK-151		2.1	37.7	GCA_002370415.1	Vegetable/Fruit/Corn/Plant	100.0	0.5			s__Leuconostoc mesenteroides	
**G18**	*L. mesenteroides*	*mesenteroides*	DRC0211		2.1	37.6	GCA_002009375.1	Vegetable/Fruit/Corn/Plant	100.0	0.5			s__Leuconostoc mesenteroides	
**G18**	*L. mesenteroides*		OG02		1.7	37.9	GCA_002276945.1	Dairy	100.0	0.5			s__Leuconostoc mesenteroides	
**G18**	*L. mesenteroides*	*mesenteroides*	LN27		2.2	37.5	GCA_002092745.1	Dairy	100.0	1.6			s__Leuconostoc mesenteroides	
**G18**	*L. mesenteroides*		SRCM102733		2.1	37.7	GCA_009913915.1	Vegetable/Fruit/Corn/Plant	100.0	0.5			s__Leuconostoc mesenteroides	
**G18**	*L. mesenteroides*		WC0331		2.0	37.6	GCA_008690745.1	Meat	100.0	1.1			s__Leuconostoc mesenteroides	
**G18**	*L. mesenteroides*	*mesenteroides*	FM06		2.0	38.0	GCA_002148235.1	Dairy	100.0	0.5			s__Leuconostoc mesenteroides	
**G18**	*Leuconostoc sp.*		DB-1		2.1	37.7	GCA_013414845.1	Vegetable/Fruit/Corn/Plant	99.0	1.1			s__Leuconostoc mesenteroides	*L. mesenteroides*
**G18**	*L. mesenteroides*		M11		2.0	37.8	GCA_009497095.1	Fish	100.0	1.6			s__Leuconostoc mesenteroides	
**G18**	*L. mesenteroides*	*mesenteroides*	LbE16		2.0	37.5	GCA_001184255.1	Dairy	100.0	1.1			s__Leuconostoc mesenteroides	
**G18**	*L. mesenteroides*	*mesenteroides*	BD1710		2.1	37.6	GCA_002117185.1	Dairy	100.0	1.1			s__Leuconostoc mesenteroides	
**G18**	***L. mesenteroides***	***mesenteroides***	**ATCC_8293**	T	2.1	37.7	GCA_000014445.1	Vegetable/Fruit/Corn/Plant	100.0	1.1	AB023242	100.0	s__Leuconostoc mesenteroides	
**G18**	*L. mesenteroides*	*mesenteroides*	LN34		1.9	37.7	GCA_002092775.1	Dairy	100.0	0.5			s__Leuconostoc mesenteroides	
**G18**	*L. mesenteroides*		WC0330		1.8	37.9	GCA_008690805.1	Meat	100.0	0.8			s__Leuconostoc mesenteroides	
**G18**	*L. mesenteroides*	*mesenteroides*	J18		2.0	37.7	GCA_000234825.3	Vegetable/Fruit/Corn/Plant	100.0	0.5			s__Leuconostoc mesenteroides	
**G18**	*L. mesenteroides*	*cremoris*	LbT16		1.9	37.8	GCA_001184265.1	Dairy	99.5	0.5			s__Leuconostoc mesenteroides	
**G18**	*L. mesenteroides*	*mesenteroides*	KMB_609		2.0	37.7	GCA_003346155.1	Dairy	100.0	1.1			s__Leuconostoc mesenteroides	
**G18**	*L. mesenteroides*	*mesenteroides*	LN25		2.3	37.5	GCA_002092735.1	Dairy	100.0	1.6			s__Leuconostoc mesenteroides	
**G18**	*L. mesenteroides*		DPC_7261		3.4	40.3	GCA_014854895.1	Vegetable/Fruit/Corn/Plant	100.0	100.0			N/A	

Group	Species	Subspecies	Strain	Type strain	Size (Mb)	GC%	Assembly	Isolation source	Completeness (%)	Contamination (%)	16S rRNA gene Genbank accession n°	16S rRNA seq. identity (%)	GTDB species cluster	Putative name *
**G18**	*L. mesenteroides*	*mesenteroides*	LN08		2.0	37.8	GCA_002092625.1	Dairy	100.0	0.5			s__Leuconostoc mesenteroides	
**G18**	*L. mesenteroides*		YL48		2.1	37.6	GCA_002886025.1	Vegetable/Fruit/Corn/Plant	100.0	0.5			s__Leuconostoc mesenteroides	
**G18**	*L. mesenteroides*		NCTC10817		2.0	37.8	GCA_900452955.1	Collection strain	100.0	1.1			s__Leuconostoc mesenteroides	
**G18**	*L. mesenteroides*	*sake*	NBRC_102481		1.7	37.8	GCA_007989505.1	Vegetable/Fruit/Corn/Plant	98.4	0.5			s__Leuconostoc mesenteroides	
**G18**	*L. mesenteroides*	*mesenteroides*	NBRC_100496		2.0	37.5	GCA_007990165.1	Vegetable/Fruit/Corn/Plant	100.0	1.1			s__Leuconostoc mesenteroides	
**G18**	*L. mesenteroides*		FDAARGOS_1033		2.1	37.7	GCA_016127295.1	Vegetable/Fruit/Corn/Plant	100.0	1.1			N/A	
**G18**	*L. mesenteroides*		CECT_9217		2.0	37.7	GCA_900290445.1	Vegetable/Fruit/Corn/Plant	100.0	0.5			s__Leuconostoc mesenteroides	
**G18**	*L. mesenteroides*		213M0		2.0	37.7	GCA_001570665.1	Dairy	99.9	1.1			s__Leuconostoc mesenteroides	
**G18**	*L. mesenteroides*	*mesenteroides*	NBRC_3832		1.9	37.6	GCA_006538765.1	Collection strain	100.0	1.1			s__Leuconostoc mesenteroides	
**G18**	*L. mesenteroides*		WC0332		1.9	37.7	GCA_008689715.1	Meat	100.0	0.5			s__Leuconostoc mesenteroides	
**G18**	*L. mesenteroides*		Wikim17		1.9	37.8	GCA_000787735.1	Vegetable/Fruit/Corn/Plant	100.0	0.8			s__Leuconostoc mesenteroides	
**G18**	*L. mesenteroides*		B5		2.1	37.5	GCA_004745755.1	Food	100.0	0.7			s__Leuconostoc mesenteroides	
**G18**	*L. mesenteroides*		FAM_18356		1.9	37.8	GCA_005864365.1	-	100.0	0.5			s__Leuconostoc mesenteroides	
**G18**	***L. mesenteroides***	***dextranicum***	**DSM_20484**	T	1.9	38.0	GCA_001047695.1	Dairy	100.0	1.3	NR_113911	100.0	s__Leuconostoc mesenteroides	
**G18**	*L. mesenteroides*	*dextranicum*	LbE15		2.0	37.6	GCA_001184245.1	Dairy	100.0	0.5			s__Leuconostoc mesenteroides	
**G18**	*L. mesenteroides*		P45		1.9	37.5	GCA_000756355.1	Vegetable/Fruit/Corn/Plant	100.0	0.0			s__Leuconostoc mesenteroides	
**G18**	*L. mesenteroides*	*mesenteroides*	LN05		2.0	38.0	GCA_002092565.1	Dairy	98.5	3.5			s__Leuconostoc mesenteroides	
**G18**	*L. mesenteroides*	*cremoris*	LMGCF01		1.6	38.0	GCA_002092045.1	Dairy	99.8	0.0			s__Leuconostoc mesenteroides	
**G18**	*L. mesenteroides*	*cremoris*	LMGCF02		1.7	37.9	GCA_002092135.1	Dairy	100.0	0.0			s__Leuconostoc mesenteroides	
**G18**	*L. mesenteroides*	*cremoris*	T26		1.8	38.4	GCA_000686485.1	Dairy	100.0	10.7			s__Leuconostoc mesenteroides	
**G18**	*L. mesenteroides*	*cremoris*	LN07		1.6	38.1	GCA_002092615.1	Dairy	100.0	0.0			s__Leuconostoc mesenteroides	
**G18**	*L. mesenteroides*	*cremoris*	LMGCF04		1.6	38.0	GCA_002092155.1	Dairy	100.0	0.0			s__Leuconostoc mesenteroides	
**G18**	*L. mesenteroides*	*cremoris*	LMGCF05		1.6	38.0	GCA_002092085.1	Dairy	100.0	0.0			s__Leuconostoc mesenteroides	
**G18**	*L. mesenteroides*	*cremoris*	TIFN8		1.7	38.2	GCA_000447945.1	Dairy	99.2	0.3			s__Leuconostoc mesenteroides	
**G18**	*L. mesenteroides*	*cremoris*	LMGCF03		1.7	38.0	GCA_002092145.1	Dairy	100.0	0.0			s__Leuconostoc mesenteroides	
**G18**	*L. mesenteroides*	*cremoris*	LMGCF17		1.6	38.0	GCA_002092425.1	Dairy	100.0	0.0			s__Leuconostoc mesenteroides	
**G18**	*L. mesenteroides*	*cremoris*	LMGCF19		1.7	38.0	GCA_002092415.1	Dairy	100.0	0.0			s__Leuconostoc mesenteroides	
**G18**	*L. mesenteroides*	*cremoris*	LMGCF14		1.6	38.0	GCA_002092335.1	Dairy	100.0	0.0			s__Leuconostoc mesenteroides	
**G18**	*L. mesenteroides*	*cremoris*	LMGCF20		1.6	38.0	GCA_002092455.1	Dairy	99.7	0.0			s__Leuconostoc mesenteroides	
**G18**	*L. mesenteroides*	*cremoris*	NBRC_107766		1.6	37.9	GCA_007992255.1	Collection strain	100.0	0.5			s__Leuconostoc mesenteroides	
**G18**	*L. mesenteroides*	*cremoris*	LMGCF16		1.6	38.0	GCA_002092515.1	Dairy	100.0	0.0			s__Leuconostoc mesenteroides	
**G18**	*L. mesenteroides*	*cremoris*	LMGCF11		1.7	38.2	GCA_002092315.1	Dairy	99.9	0.0			s__Leuconostoc mesenteroides	
**G18**	*L. mesenteroides*	*cremoris*	LMGCF10		1.6	38.0	GCA_002092245.1	Dairy	100.0	0.0			s__Leuconostoc mesenteroides	
**G18**	*L. mesenteroides*	*cremoris*	LMGCF12		1.7	38.1	GCA_002092385.1	Dairy	100.0	0.5			s__Leuconostoc mesenteroides	
**G18**	*L. mesenteroides*	*cremoris*	LMGCF13		1.6	38.3	GCA_002092465.1	Dairy	100.0	1.1			s__Leuconostoc mesenteroides	
**G18**	*L. mesenteroides*		MGYG-HGUT-01341		1.7	38.6	GCA_902374135.1	Human sample	100.0	0.0			s__Leuconostoc mesenteroides	
**G18**	***L. mesenteroides***	***cremoris***	**ATCC_19254**	T	1.7	38.5	GCA_000160595.1	Dairy	100.0	0.0	NR_040818	100.0	s__Leuconostoc mesenteroides	
**G18**	*L. mesenteroides*	*cremoris*	LMGCF18		1.6	38.0	GCA_002092495.1	Dairy	100.0	0.0			s__Leuconostoc mesenteroides	
**G18**	*L. mesenteroides*	*cremoris*	LMGCF07		1.6	38.1	GCA_002092215.1	Dairy	100.0	0.0			s__Leuconostoc mesenteroides	
**G18**	*L. mesenteroides*	*cremoris*	LMGCF09		1.6	38.1	GCA_002092105.1	Dairy	100.0	0.0			s__Leuconostoc mesenteroides	
**G18**	*L. mesenteroides*		TR154		1.9	37.8	GCA_006406185.1	Vegetable/Fruit/Corn/Plant	100.0	0.5			s__Leuconostoc mesenteroides	
**G18**	*L. mesenteroides*		CBA7131		2.1	37.6	GCA_003255835.1	Human sample	99.5	0.5			s__Leuconostoc mesenteroides	
**G18**	*L. mesenteroides*	*mesenteroides*	BD3749		2.0	37.8	GCA_001583825.1	Vegetable/Fruit/Corn/Plant	100.0	0.5			s__Leuconostoc mesenteroides	

Group	Species	Subspecies	Strain	Type strain	Size (Mb)	GC%	Assembly	Isolation source	Completeness (%)	Contamination (%)	16S rRNA gene Genbank accession n°	16S rRNA seq. identity (%)	GTDB species cluster	Putative name *
**G18**	*L. mesenteroides*		SRCM103453		2.2	37.6	GCA_004103675.1	Food	100.0	0.5			s__Leuconostoc mesenteroides	
**G18**	*L. mesenteroides*		SRCM102735		2.1	37.6	GCA_009913935.1	Vegetable/Fruit/Corn/Plant	100.0	0.5			s__Leuconostoc mesenteroides	
**G18**	*L. mesenteroides*		SRCM103356		2.0	37.6	GCA_004102585.1	Food	100.0	0.7			s__Leuconostoc mesenteroides	
**G18**	***L. mesenteroides***	***jonggajibkimchii***	**DRC1506**	T	2.0	37.6	GCA_001886915.1	Vegetable/Fruit/Corn/Plant	99.9	1.1	NR_157602	100.0	s__Leuconostoc mesenteroides	
**G18**	*L. mesenteroides*	*mesenteroides*	KIBGE-IB22		2.0	37.5	GCA_005049065.1	Vegetable/Fruit/Corn/Plant	100.0	0.7			s__Leuconostoc mesenteroides	
**G18**	*L. mesenteroides*		KFRI-MG		1.9	37.7	GCA_000512955.1	Vegetable/Fruit/Corn/Plant	100.0	0.5			s__Leuconostoc mesenteroides	
**G18**	*L. mesenteroides*		WiKim33		2.0	37.6	GCA_003433375.1	Vegetable/Fruit/Corn/Plant	100.0	0.5			s__Leuconostoc mesenteroides	
**G18**	*L. mesenteroides*		SG0315		2.1	37.5	GCA_014060975.1	Vegetable/Fruit/Corn/Plant	100.0	1.1			s__Leuconostoc mesenteroides	
**G18**	*L. mesenteroides*		SRCM103460		2.1	37.6	GCA_004194375.1	Food	100.0	0.5			s__Leuconostoc mesenteroides	
**G18**	*L. mesenteroides*		WC0333		2.1	37.5	GCA_008689685.1	Meat	100.0	0.5			s__Leuconostoc mesenteroides	
**G18**	*L. mesenteroides*		406		2.0	37.7	GCA_001515385.1	Dairy	100.0	1.6			s__Leuconostoc mesenteroides	

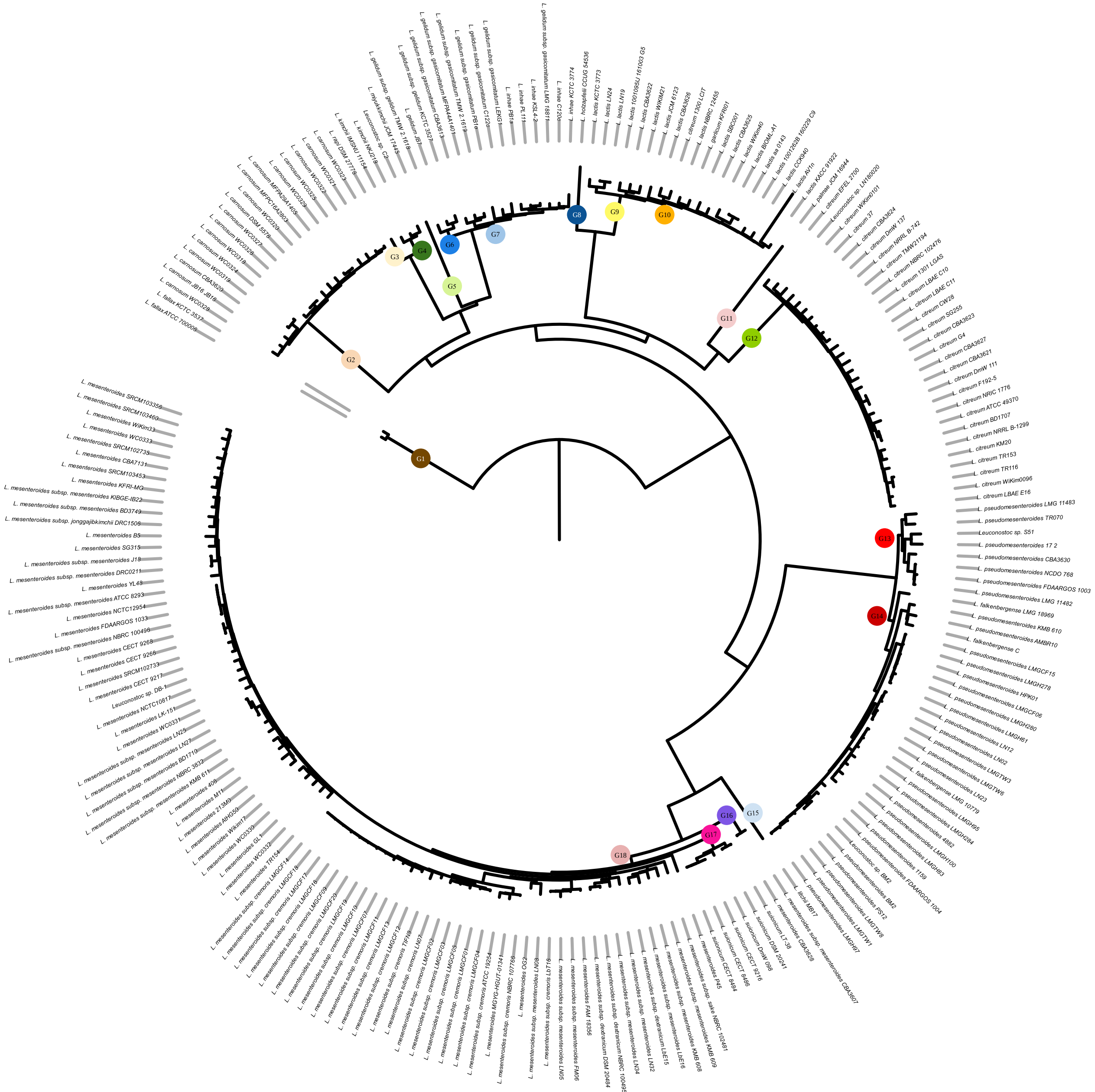

Supplementary figure 1: ANI (A), core genome (B), AAI (C), *rpoA* gene (D), *pheS* gene (E), and 16S rRNA gene (F) trees. * for clarity of representation, the branch of the strain AV1N in the *pheS* tree was shorted due to high sequence divergence.

For core genome, *rpoA*, *pheS* and 16S rRNA genes trees, an alignment was produced with Clustal Omega respectively and it was used to infer a tree using RAxML. ANI tree was inferred from ANI distance matrix with R package APE. AAI distance matrix was used to compute the UPGMA tree.

Number of nucleotide positions analyzed for each alignment: core genome, 236,838; *rpoA*, 945; *pheS*, 1,104; 16S rRNA, 1567.

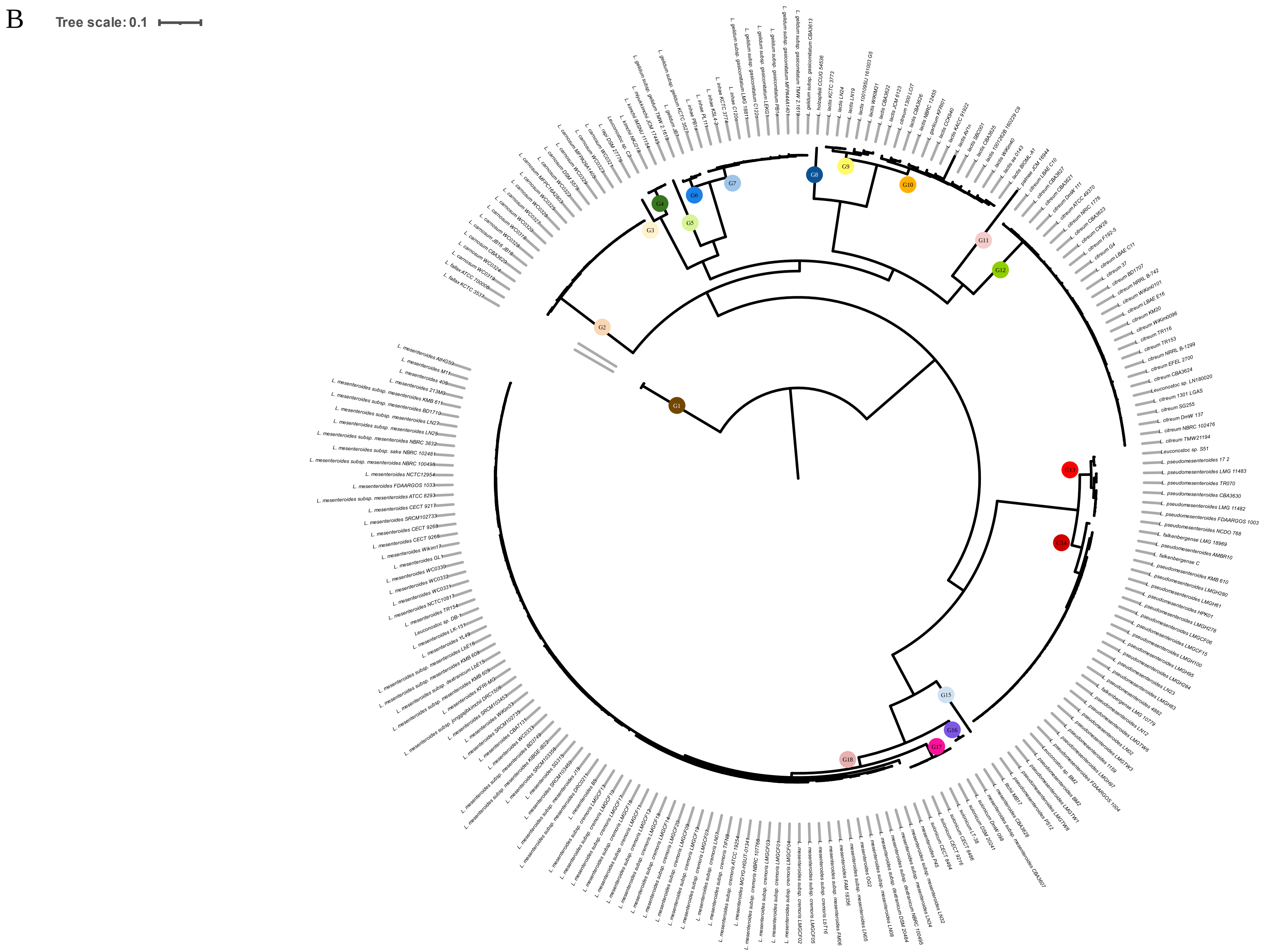
B
Tree scale: 0.1
G1
G2
G3
G4
G5
G6
G7
G8
G9
G10
G11
G12
G13
G14
G15
G16
G17
G18

C

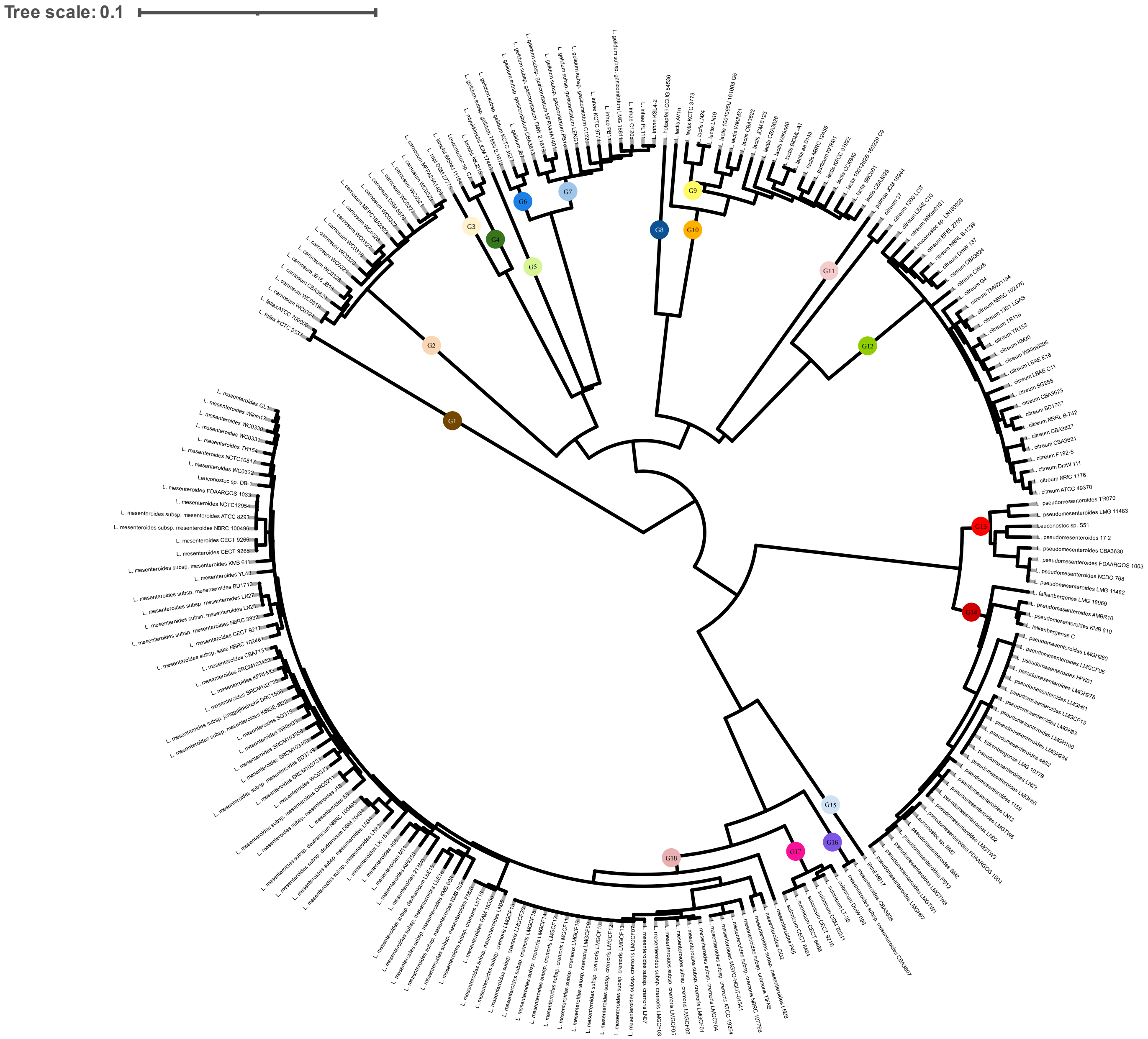

Tree scale: 0.1
G1
G2
G3
G4
G5
G6
G7
G8
G9
G10
G11
G12
G13
G14
G15
G16
G17
G18

D

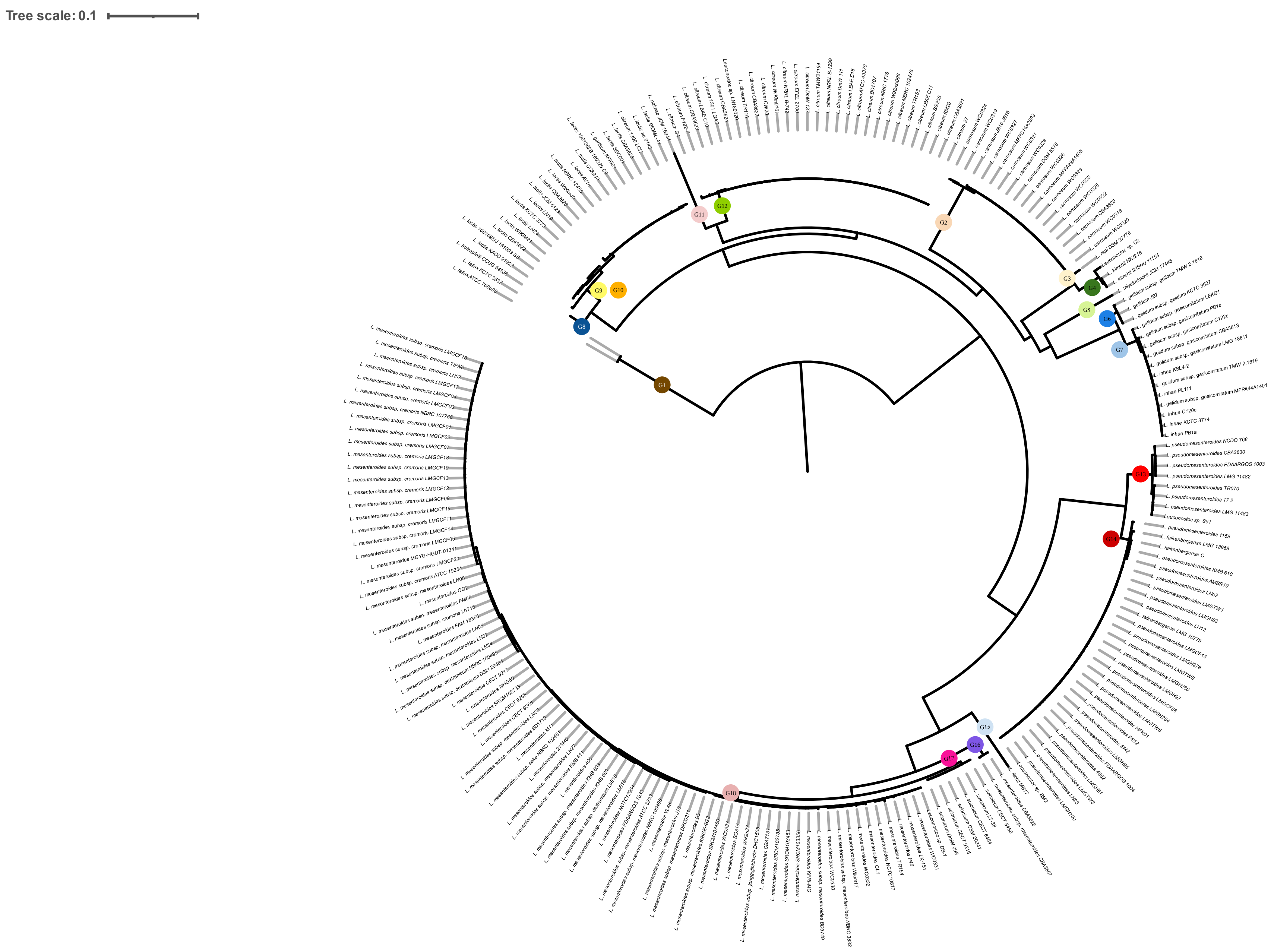
Tree scale: 0.1
G1
G2
G3
G4
G5
G6
G7
G8
G9
G10
G11
G12
G13
G14
G15
G16
G17
G18

E

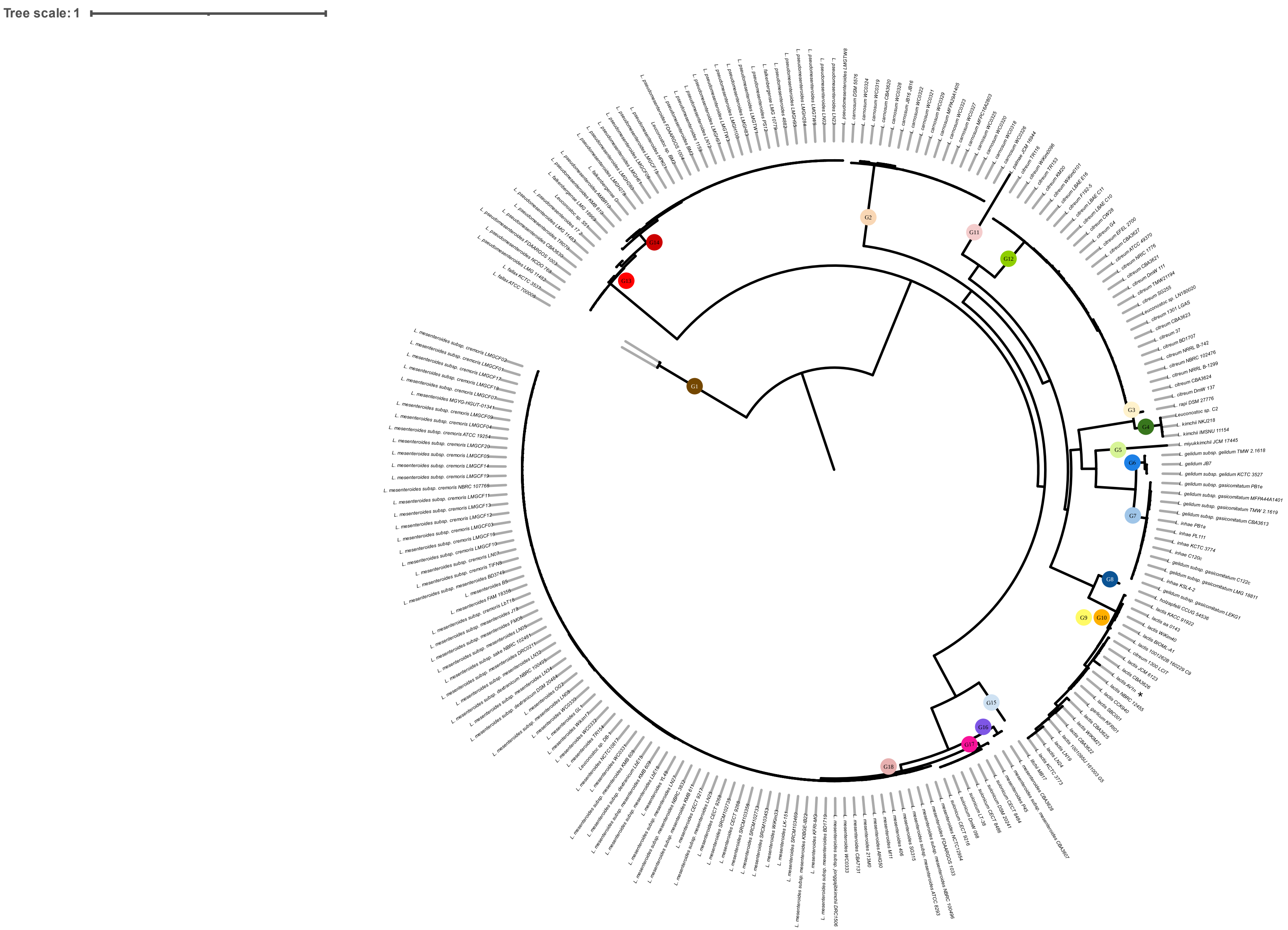
Tree scale: 1
G1
G2
G3
G4
G5
G6
G7
G8
G9
G10
G11
G12
G13
G14
G15
G16
G17
G18

F

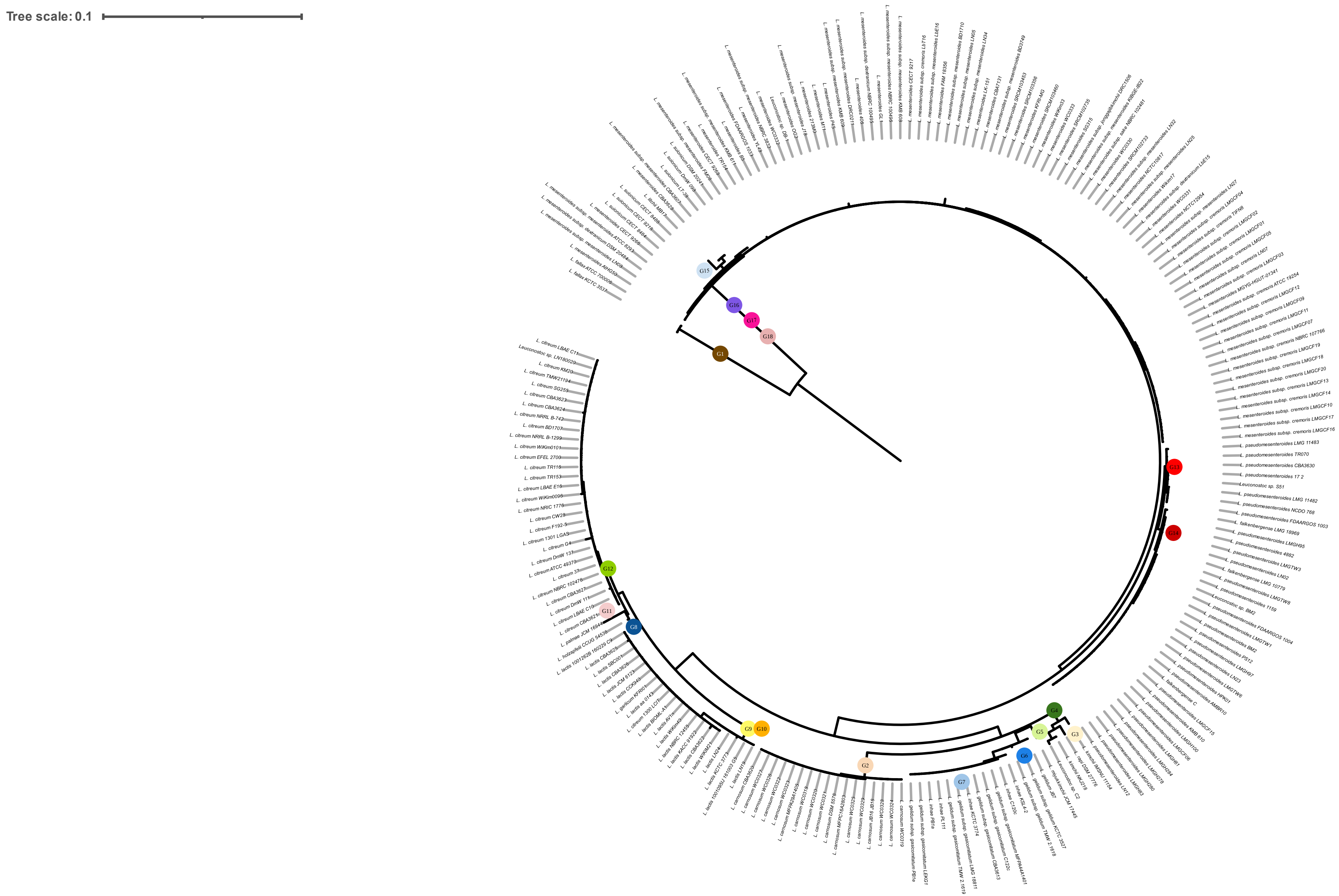

Tree scale: 0.1
G1
G2
G3
G4
G5
G6
G7
G8
G9
G10
G11
G12
G13
G14
G15
G16
G17
G18

Supplementary figure 2: ANI (A), core genome (B), *rpoA* gene (C), *pheS* gene (D), and 16 rRNA gene (E) trees reporting bootstrap values. Branch length is ignored for clarity.

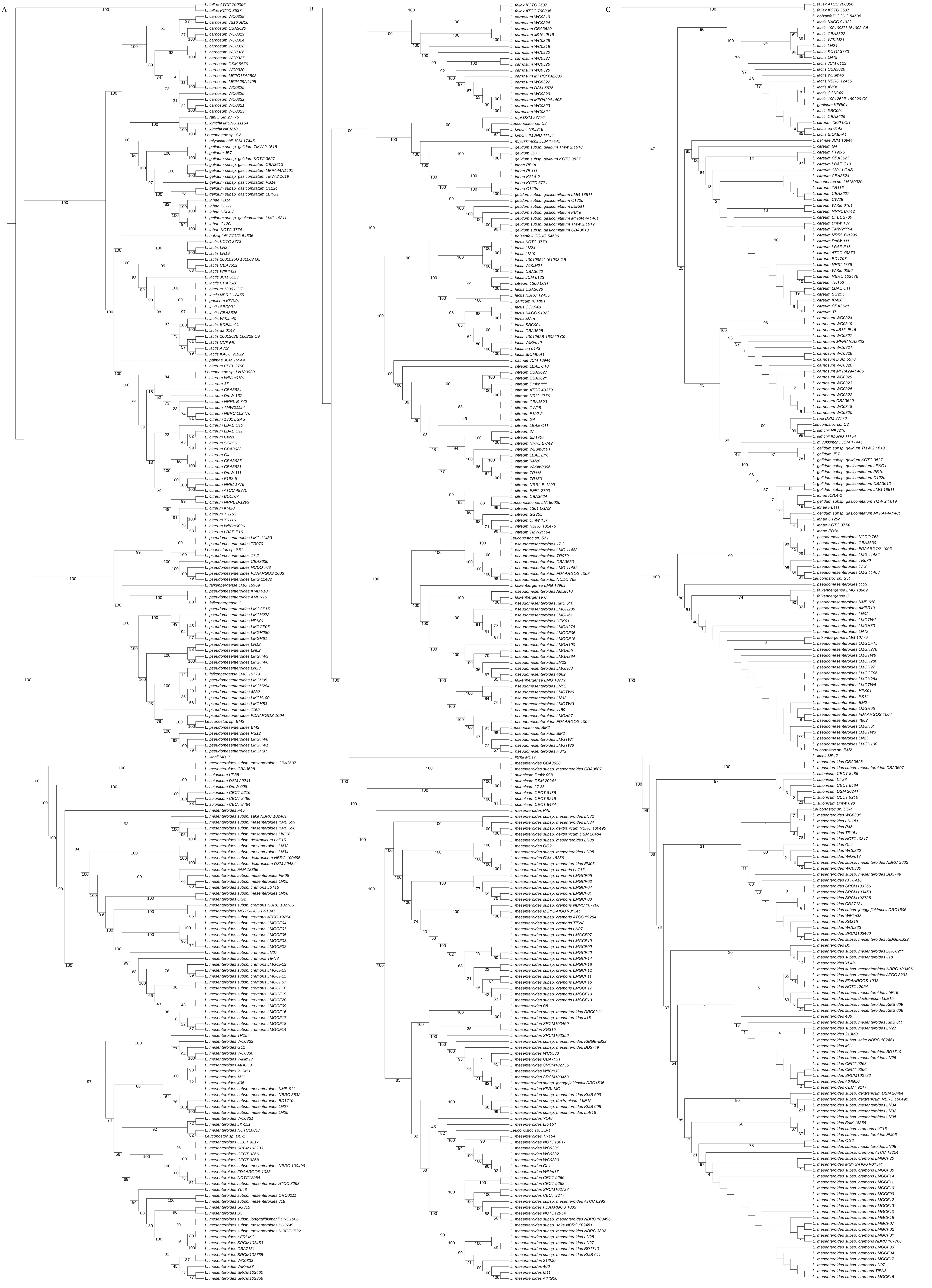

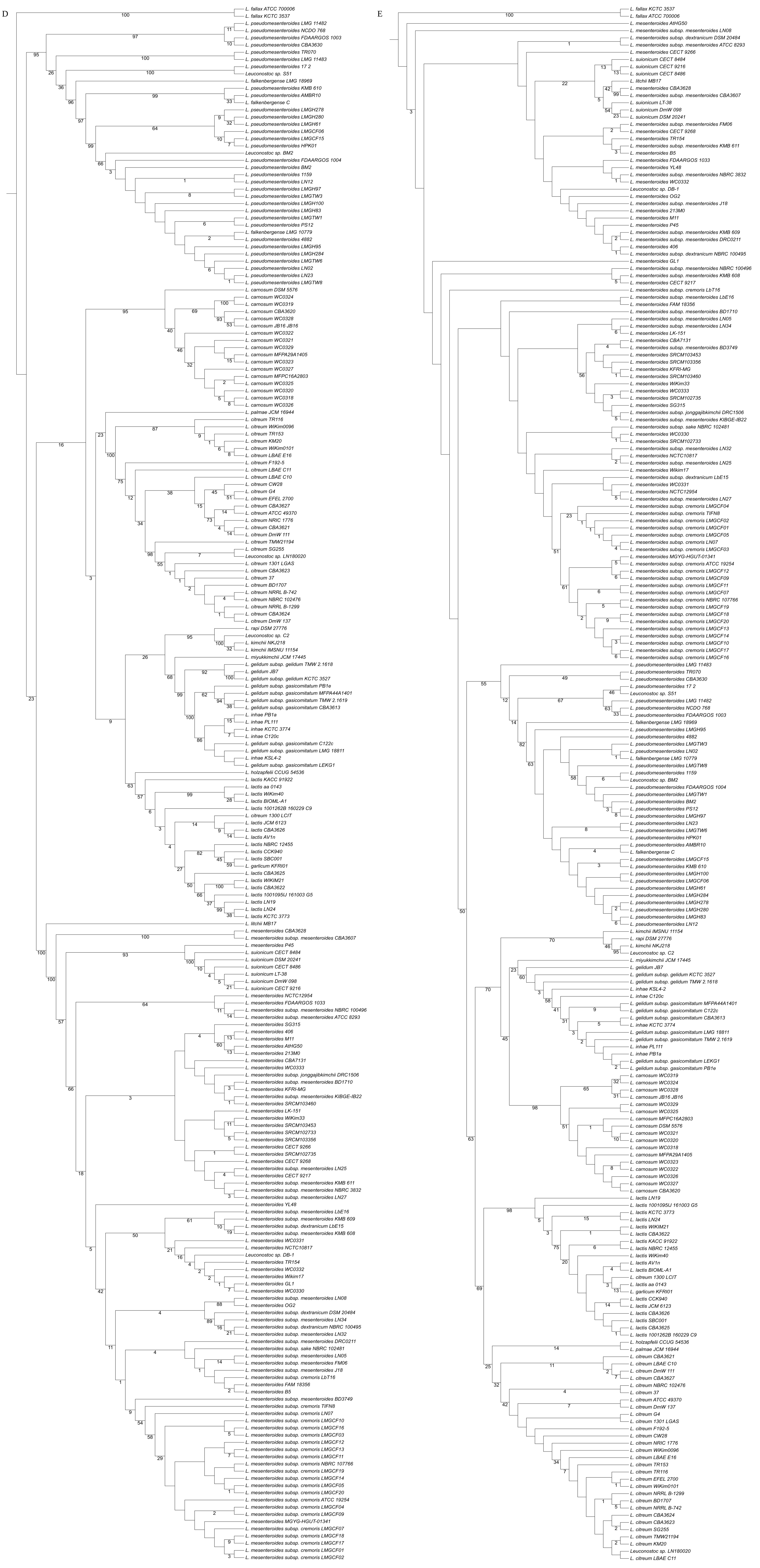
D
E
